# Bcl-2 supports survival and metabolic fitness of quiescent tissue-resident ILC3

**DOI:** 10.1016/j.mucimm.2023.07.001

**Published:** 2023-10

**Authors:** James I. King, Felipe Melo-Gonzalez, Bert Malengier-Devlies, Roser Tachó-Piñot, Marlene S Magalhaes, Suzanne H. Hodge, Xavier Romero Ros, Rebecca Gentek, Matthew R. Hepworth

**Affiliations:** 1 Lydia Becker Institute of Immunology and Inflammation, University of Manchester, Manchester, United Kingdom; 2 Division of Immunology, Immunity to Infection and Respiratory Medicine, School of Biological Sciences, Faculty of Biology, Medicine and Health, Manchester Academic Health Science Centre, University of Manchester, Manchester, United Kingdom; 3 Institute for Regeneration and Repair, Centre for Inflammation Research & Centre for Reproductive Health, University of Edinburgh, Edinburgh, United Kingdom; 4 Bioscience Asthma, Research and Early Development, Respiratory & Immunology, BioPharmaceuticals R&D, AstraZeneca, Cambridge, United Kingdom

## Abstract

Group 3 innate lymphoid cells (ILC3) are potent effector cells with critical roles in enforcing immunity, barrier integrity and tissue homeostasis along the gastrointestinal tract. ILC3 are considered primarily tissue-resident cells, seeding the gastrointestinal tract during embryonic stages and early life. However, the mechanisms through which mature ILC3 are maintained within adult tissues are poorly understood. Here, we report that lymphoid tissue-inducer-like (LTi-like) ILC3 exhibit minimal turnover in the healthy adult intestinal tract, persist for extended periods of time, and display a quiescent phenotype. Strikingly, during enteric bacterial infection LTi-like ILC3 also exhibit negligible hematopoietic replenishment and remain non-proliferative, despite robustly producing cytokines. Survival of LTi-like ILC3 was found to be dependent upon the balance between the metabolic activity required to drive effector function and anti-apoptotic programs. Notably, the pro-survival protein B-cell lymphoma-2 (Bcl-2) was required for the survival of LTi-like ILC3 *ex vivo* but was rendered partially dispensable if mitochondrial respiration was inhibited. Together we demonstrate LTi-like ILC3 are a tissue-resident, quiescent population that persist independently of hematopoietic replenishment to survive within the intestinal microenvironment.

## INTRODUCTION

The gastrointestinal tract represents a highly dynamic environment in which a layered immune system acts to maintain tissue health and homeostatic function[1], [2], [3]. In response to continual challenge and stimulus, many intestinal immune cell populations turnover rapidly and are reliant on continued replenishment from circulating bone marrow-derived progeny[4], [5], [6]. In contrast, the turnover of mature innate lymphoid cells (ILC) within tissue microenvironments remains incompletely defined. ILCs are increasingly thought to be primarily tissue-resident and non-circulatory, with the exception of natural killer (NK) cells and ILC1[7], [8]. In contrast, migration between the gut and organized intestinal-associated lymphoid tissues can occur via the lymphatics, suggesting ILC may indeed leave the tissue and enter other organs in some contexts[9], [10]. Group 3 innate lymphoid cells (ILC3), characterized by expression of retinoic acid-related orphan receptor gamma (RORγt), are among the first lymphocyte populations to establish residency in the gut and play important roles in driving lymphoid tissue organogenesis, and in maintaining intestinal barrier health throughout the life course via constitutive production of the cytokine interleukin (IL)-22^11^. Recent advances have further defined the early ontological events that determine ILC3 seeding prior to birth and during the neonatal period[12], [13], [14]. However, the mechanisms which intestine-resident ILC3 utilize to persist throughout adulthood, and the extent to which the bone marrow contributes to maintenance of this population remains poorly understood.

Intestinal ILC3 exhibits extensive heterogeneity and comprise at least three distinct subsets with significant differences in tissue localization, transcriptional regulation, cell surface phenotype and biological functions^15^. Specifically, ILC3 can be split into CCR6^+^ lymphoid tissue-inducer-like (LTi-like) ILC3, natural cytotoxicity receptor-expressing (NCR^+^) ILC3 and NCR^−^ CCR6^−^ “double negative” (DN) ILC3, all of which have the capacity to mediate protective effector responses to extracellular microbes via the production of IL-22^15^. Despite sharing overlapping effector cytokine profiles, ILC3 subsets exhibit the capacity to perform vastly divergent functions. LTi-like ILC3 are localized specifically within lymph nodes and intestinal-associated lymphoid structures[9], [16], [17], [18] and possess a variety of regulatory functions that act to modulate adaptive immunity at mucosal barrier surfaces^19^. In contrast, NCR^+^ ILC3 are typically dispersed within the intestinal lamina propria and establish residence only following microbial colonization post-birth[14], [20], [21]. Unlike LTi-like ILC3, NCR^+^ ILC3 and DN ILC3 co-express the transcription factor T-bet which confers the capacity to secrete interferon-γ, and in inflammatory contexts NCR^+^ ILC3 can lose RORγt expression and undergo conversion to an ‘ex-ILC3’ or ILC1-like pro-inflammatory phenotype[22], [23], [24]. Despite this divergent biology, ILC3 subset-specific differences are often overlooked, and as a result ILC3 have been attributed seemingly contradictory roles in the context of intestinal immunity and inflammatory disease. Together this provokes the need for a better understanding of the differences in ILC3 subset-specific biology in health and disease.

Here we demonstrate intestinal ILC3 are long-lived tissue-resident cells that display relatively little hematopoietic replenishment under steady-state conditions. We report LTi-like ILC3 exhibit a quiescent-like phenotype *in vivo* and are reliant on the anti-apoptotic protein B-cell lymphoma 2 (Bcl-2) *ex vivo* to facilitate survival—in part by protecting cells against oxidative phosphorylation (OXPHOS)-mediated metabolic stress. Together these findings highlight LTi-like ILC3 as long-lived tissue-resident effector cells adapted for persistence within their tissue niche. Moreover, these data further define the contrasting biology underpinning the immune functions of ILC3 subsets within mucosal barrier tissues—which may shed light on their roles in inflammatory diseases, such as Inflammatory Bowel Disease (IBD).

## RESULTS

### Intestinal ILC3s are maintained independent of bone marrow replenishment

Previous studies have demonstrated that mature ILC do not typically recirculate in the blood^7^, suggesting they may be maintained within tissues through mechanisms that remain unclear. To further define the turnover of ILC3 subsets within the intestine and associated lymphoid structures, we first utilized a previously described tamoxifen-inducible fate-mapping model under the control of the *Id2* allele, a transcription factor common to all ILCs (*Id2*^CreERT2–RFP^)[25], [26] (representative gating strategies; Supplementary Fig. S1), to label intestinal ILC. Eight-week-old *Id2*^CreERT2-RFP^ mice received five doses of tamoxifen via oral gavage over the course of 10 days and were subsequently assessed either 10 days post-treatment or 6 weeks later (day 52). The frequencies of RFP^+^ cells among both NCR^+^ and LTi-like ILC3 subsets were observed to decrease only slightly over a 6-week period, with the vast majority of RFP^+^ cells surprisingly maintained—suggestive of a relatively low turnover of ILC3 (Supplementary Figs. S2A and S2B).

**Fig. 1:**
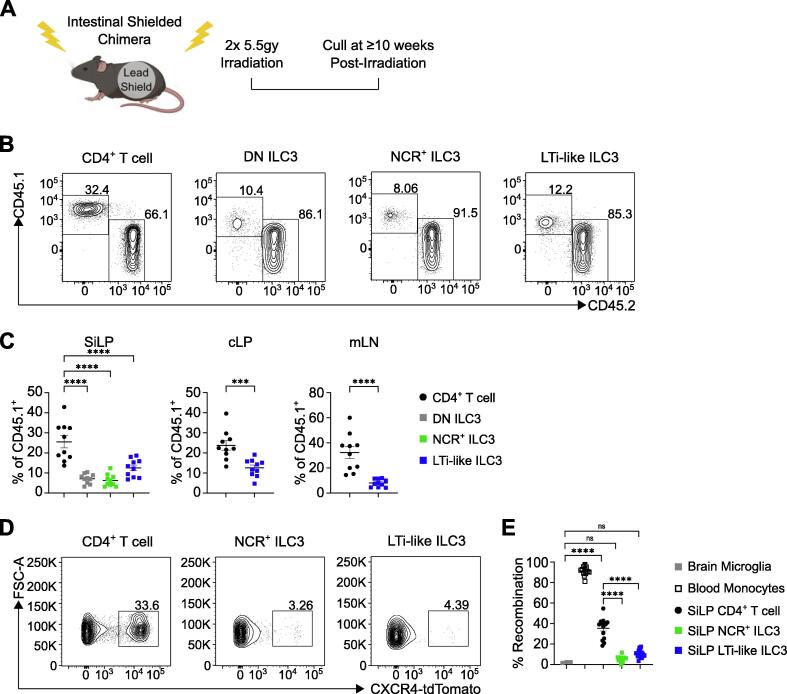
Intestinal ILC3s are maintained independently of bone marrow replenishment. (A) Experimental design for intestinal shielded bone marrow chimeras. Recipient (CD45.2^+^) adult C57BL/6 mice received sub-lethal whole-body irradiation with a lead shield covering the intestines followed by injection of donor (CD45.1^+^) bone marrow. Mice were culled ≥ 10 weeks post-irradiation. (B) Representative flow cytometry plots of CD45.1^+^ and CD45.2^+^ CD4^+^ T cells, DN ILC3, NCR^+^ ILC3 and LTi-like ILC3 in SiLP at ≥ 10 weeks post-irradiation. (C) Frequency of CD45.1^+^ CD4^+^ T cells, DN ILC3, NCR^+^ ILC3 and LTi-like ILC3 in SiLP pooled from three independent experiments at >10 weeks post-irradiation. (D and E) Frequency of Cxcr4-^CreERT2-tdTomato^ labeled blood monocytes (CD11b^+^, Ly6C^+^), microglia (CD11b^+^, CD64^+^, F4/80^+^), CD4^+^ T cells, NCR^+^ ILC3 and LTi-like ILC3 12 weeks post-labeling (Microglia, CD4^+^ T cell and ILC3 are normalized to blood monocytes). Panel B-C gated as in Supplementary Fig. S1A, panels D–E gated as in Supplementary Fig. S1B. Data shown as mean ± standard error of mean and represents two (Microglia in E) or three independent experiments (*n* = 10–14). Numbers in flow plot indicate percentage of cells in the respective gate. * *p* < 0.05, ** *p* < 0.01, *** *p* < 0.001, **** *p* < 0.0001 using unpaired t test or Tukey’s (C) or Holm-Šídák’s (E) multiple comparisons test. CD = clusters of differentiation; DN = double negative; ILC3 = group 3 innate lymphoid cell; LTi-like = lymphoid tissue-inducer-like; mLN = mesenteric lymph nodes; NCR = natural cytotoxicity receptor-expressing; ns = not significant.

Many peripheral immune cell populations are continually replenished over the life course via bone marrow hematopoiesis. To address the bone marrow contribution toward the intestinal ILC compartment in adults, we generated bone marrow chimeras in which the gastrointestinal tract was shielded to protect tissue-resident cells and analyzed the contribution of congenic donor bone marrow (CD45.2^+^) to intestinal lymphocyte populations (CD45.1^+^) at least 10 weeks post-irradiation (Fig. 1A). Notably, while other tissue-resident lymphocytes including CD4^+^ T cells exhibited approximately 30% chimerism over this time period we found limited chimerism among ILC3 subsets, including LTi-like ILC3, NCR^+^ ILC3, and DN ILC3 in the small intestine (Figs. 1B and 1C), as well as the colon and mesenteric lymph nodes (Fig. 1C). These findings suggest adult ILC3 populations exhibit a relatively low input from bone marrow hematopoiesis when compared to other lymphocyte populations at steady state. Nonetheless, to determine whether bone marrow replenishment could occur if the host intestinal ILC3 compartment was first radio-ablated, we exposed mice to sub-lethal irradiation in the absence of intestinal shielding (Supplementary Fig. S2C). In this context bone marrow-mediated replenishment of the ILC3 compartment was significantly elevated (Supplementary Figs. S2D and S2E), indicating the bone marrow retains the capacity to replenish intestinal ILC3 subsets following significant disruption of the cell population and/or tissue damage, but that this does not typically occur in otherwise healthy animals (Figs. 1A–C). Together these data suggest intestinal ILC3 populations may be long-lived tissue-resident cells at homeostasis.

To circumvent confounding factors associated with exposing mice to irradiation, we further tested the contribution of adult bone marrow hematopoiesis to ILC3 replenishment by using a tamoxifen-inducible *Cxcr4*^ERT2Cre-tdTomato^ fate-mapping model, which induces tdTomato expression in hematopoietic stem cells upon tamoxifen administration and subsequently marks arising progeny^27^. Adult mice were tamoxifen-treated and culled 12 weeks post-labeling. In line with shielded chimera data, a comparable frequency of tdTomato^+^ cells was observed among small intestinal lamina propria (SiLP) CD4^+^ T cells (Figs. 1D and 1E), further indicating bone marrow contribution to intestinal T cell replenishment over this timeframe. In contrast, ILC3 subsets had only negligible frequencies of tdTomato^+^ cells (<10%), a rate of recombination that was comparable with other long-lived tissue-resident cell populations, including microglia in the brain (Figs. 1D and 1E). As expected, circulating blood monocytes exhibited near-complete tdTomato labeling—in line with the rapid turnover and bone marrow replenishment of this population (Fig. 1E). Overall, these data suggest mature intestinal ILC3s are subject to minimal replenishment from the bone marrow during adulthood and are long-lived tissue-resident cells.

### LTi-like ILC3 are non-proliferative quiescent-like effector lymphocytes

**Fig. 2:**
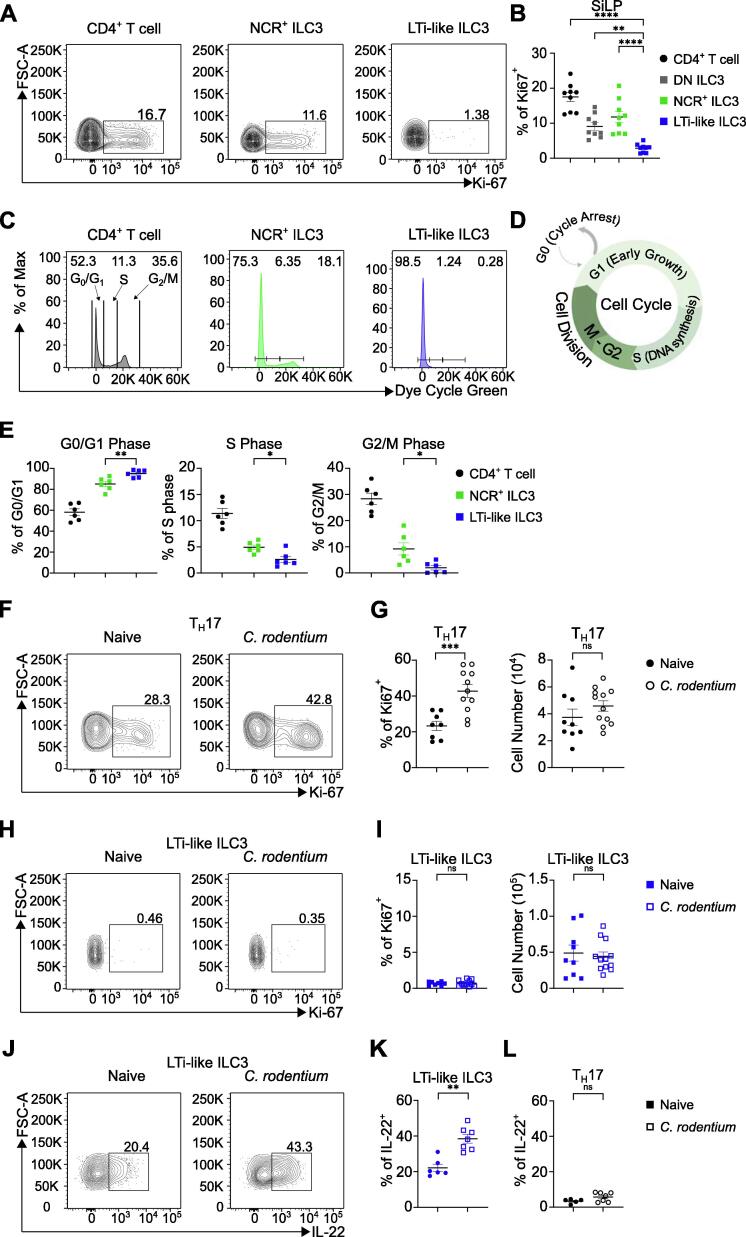
Distinct proliferative capacity of intestinal ILC3 subsets. (A and B) Frequency of Ki-67 expression in CD4^+^ T cells, DN ILC3, NCR^+^ ILC3 and LTi-like ILC3 in SiLP. (C) Representative histogram of G0/G1 phase, S phase and G2/M phase of CD4^+^ T cells, NCR^+^ ILC3 and LTi-like ILC3 in SiLP using a cell cycle dye. (D) Cartoon outlining the key cell cycle stages from cycle arrest to mitosis. (E) Frequency of G0/G1 phase, S phase and G2/M phase of CD4^+^ T cells, NCR^+^ ILC3 and LTi-like ILC3 in SiLP using a cell cycle dye. (F and G) Frequency of Ki-67 expression and cell number in T_H_17 cells in cLP of naïve or C. rodentium infected (day 6) mice. (H and I) Frequency of Ki-67 expression and cell number in LTi-like ILC3 in cLP of naïve or *Citrobacter rodentium* infected (day 6) mice. (J–L) Frequency of interleukin-22 production in LTi-like ILC3 and T_H_17 cells in cLP of naïve or *C. rodentium* infected (day 6) mice. CD4^+^ T cell, T_H_17 cell and ILC3 subsets in panels A–B and F–L gated as in Supplementary Fig. S1A, panels 2C–E gated as in Supplementary Fig. S1B. Data shown as mean ± standard error of mean and represent two (E) or three (B, G, I, K, L) independent experiments (*n* = 6–11). Numbers in flow plot indicate percentage of cells in the respective gate. * *p* < 0.05, ** *p* < 0.01, *** *p* < 0.001, **** *p* < 0.0001 using unpaired t test (B, G) or Mann–Whitney test (E, I, K, L). CD = clusters of differentiation; DN = double negative; ILC3 = group 3 innate lymphoid cell; LTi-like = lymphoid tissue-inducer-like; NCR = natural cytotoxicity receptor-expressing; ns = not significant; T_H_17 = T helper 17.

The relatively low contribution of bone marrow input to homeostatic ILC3 maintenance provoked the question of how tissue-resident ILC3s are maintained in the adult intestine. One way in which immune cell populations may be replenished within tissues is via proliferative self-renewal. Thus, we determined the proliferative status of intestinal ILC3s at homeostasis by measuring the expression levels of the marker Ki-67. Strikingly, we noted a clear difference in the frequency of Ki-67 expressing cells between lymphocyte subsets, with a significant proportion of CD4^+^ T cells, DN ILC3 and NCR^+^ ILC3 exhibiting Ki-67 expression (Figs. 2A and 2B). Similarly, we observed steady-state levels of proliferation in colonic ILC2 and CD4^+^ T cells (Supplementary Figs. S3A and S3B). Surprisingly, LTi-like ILC3 exhibited negligible to undetectable expression of Ki-67 (<2%) in both the small and large intestine (Figs. 2A and 2B; Supplementary Figs. S3A and S3B). To further assess the proliferative status of ILC3 subsets, we used a cell cycle dye (Figs. 2C and 2D). CD4^+^ T cells and NCR^+^ ILC3 exhibited elevated cell cycle dye staining indicative of cells in the S phase and the G2/M phase stages of the cell cycle, reflecting cell cycle progression toward mitosis (Figs. 2C–E). Comparatively, LTi-like ILC3 had uniformly low staining for the dye (Figs. 2C–E), suggesting very little progression through cell cycle or cell division, further indicating LTi-like ILC3 are largely non-proliferative at steady state. Thus, together our findings suggest LTi-like ILC3 are a quiescent-like population under homeostatic conditions.

We next determined whether LTi-like ILC3 engages proliferation to maintain or expand cell numbers following a physiologically relevant stimulus. To assess this we infected mice with the enteric pathogen *Citrobacter rodentium* which has been extensively demonstrated to elicit potent effector cytokine responses from ILC3, and which specifically requires LTi-like ILC3-derived IL-22 for optimal protective immunity[20], [28], [29], [30]. As a point of comparison we assessed the proliferation of T helper 17 (T_H_17) cells*.* As expected, we observed a significant increase in the proliferation of colonic T_H_17 cells following *C. rodentium* infection (Figs. 2F and 2G). Intriguingly however, we saw no change in the Ki-67 expression of LTi-like ILC3 at day 6 of *C. rodentium* infection in the colon (Figs. 2H and 2I; Supplementary Figs. S3C and S3D), despite exhibiting a robust increase in IL-22 production in response to infection as expected (Figs. 2J and 2K; Supplementary Figs. S3E and S3F). In comparison, T_H_17 cells did not yet produce significant amounts of IL-22 at this early time point of infection (Fig. 2L). To determine whether hematopoietic replenishment of ILC3 occurred in the context of enteric infection and elevated effector function, we infected tamoxifen-treated *Cxcr4*^ERT2Cre-tdTomato^ mice with *C. rodentium* and assessed bone marrow contribution to the ILC3 compartment following resolution of infection (Supplementary Fig. S4). Blood monocytes were uniformly labeled by tdTomato regardless of infection status, confirming the high turnover and constitutive hematopoietic replenishment of circulating myeloid cells (Supplementary Fig. S4A). Within the colon tissue of infected animals, we detected an increase in infiltrating CD11b^Hi^ cells, which notably expressed elevated levels of tdTomato—likely reflecting an elevated contribution of hematopoiesis to support the infiltrating myeloid cell compartment (Supplementary Figs. S4B–D). Despite these observations, ILC3 subsets did not exhibit any increase in tdTomato expression in response to *C. rodentium*, indicating that even following infection, bone marrow contribution to the maintenance of intestinal ILC3 populations remains limited (Supplementary Figs. S4E and S4F). Together these results reveal that LTi-like ILC3 remains quiescent-like and non-proliferative despite mediating innate effector responses to *C. rodentium* infection.

### Proliferation induced by supraphysiological stimulus depletes LTi-like ILC3, but not other ILC subsets

**Fig. 3:**
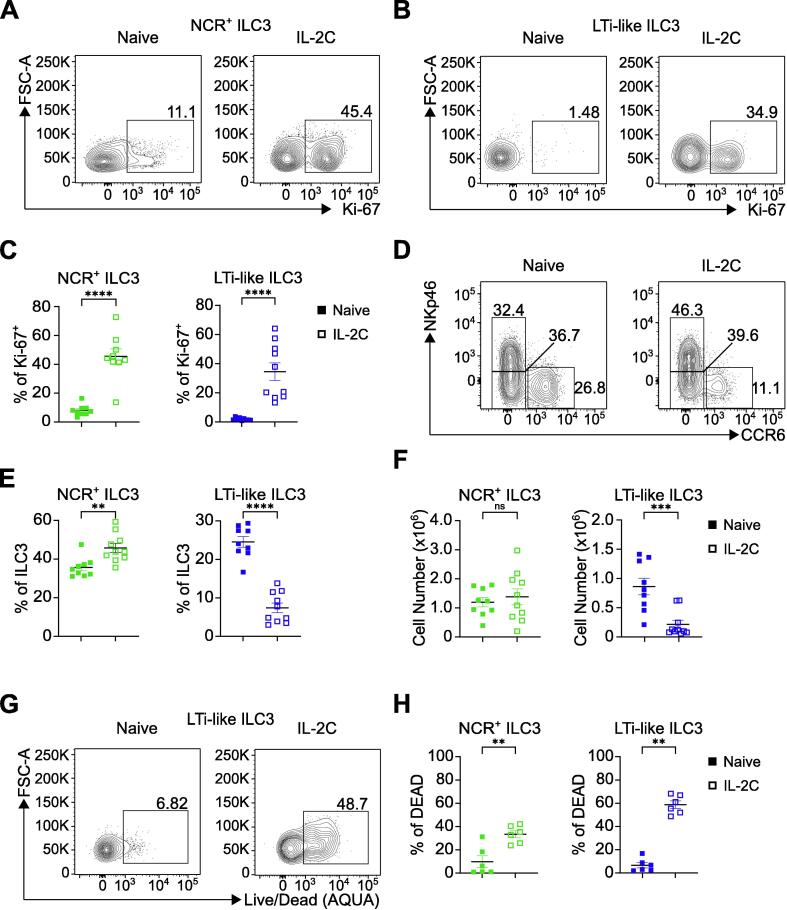
IL-2C-induced proliferation results in depletion of LTi-like ILC3. (A–C) Frequency of Ki-67 expression in NCR^+^ ILC3 and LTi-like ILC3 in SiLP in naïve or IL-2C treated mice. (D–F) Frequency and cell number of NCR^+^ ILC3 and LTi-like ILC3 in SiLP in naïve or IL-2C treated mice. (G and H) Frequency of dead NCR^+^ ILC3 and LTi-like ILC3 in SiLP in naïve or IL-2C treated mice. ILC3 subsets identified via gating strategy in Supplementary Fig. S1A. Data shown as mean ± standard error of mean and represent three independent experiments (*n* = 6–10). Numbers in flow plot indicate percentage of cells in the respective gate. * *p* < 0.05, ** *p* < 0.01, *** *p* < 0.001, **** *p* < 0.0001 using Mann–Whitney test. ILC3 = group 3 innate lymphoid cell; IL-2C = interleukin-2 complex; LTi-like = lymphoid tissue-inducer-like; ns = not significant; NCR = natural cytotoxicity receptor-expressing.

As LTi-like ILC3s did not proliferate at steady state or in response to *C. rodentium* infection, we next sought to understand whether LTi-like ILC3 possessed the capacity to proliferate if exposed to a supraphysiological stimulus. LTi-like ILC3 express high levels of the IL-2Ra subunit CD25 suggesting they are responsive to this classically proliferative cytokine^31^. Thus, we treated mice *in vivo* with IL-2 complex (IL-2C), which has been shown to promote high levels of proliferation in skin ILCs^32^. Following IL-2C treatment we saw a striking increase in the proliferation of ILC2, NCR^+^ ILC3 and DN ILC3 as expected, but also of LTi-like ILC3—indicating that resident LTi-like ILC3 retain the capacity to proliferate in response to significant stimulation (Figs. 3A–C; Supplementary Figs. S5A–E). Increased proliferation correlated with increases in NCR^+^ ILC3, DN ILC3 and ILC2 relative cell frequency as expected (Supplementary Fig. S5F). However, we saw a surprising decrease in LTi-like ILC3 both as a frequency of ILC3 and as a frequency of CD45^+^ cells (Figs. 3D and 3E; Supplementary Fig. S5F). IL-2C treatment led to notable macroscopic changes in intestinal tissue that impacted the efficiency of our enzymatic digest and consistently reduced small intestinal cell yields. Nonetheless, only total cell numbers of LTi-like ILC3, but not other ILC populations, were significantly reduced following IL-2C treatment (Fig. 3F; Supplementary Fig. S5G), which was associated with a significant proportion of LTi-like ILC3 staining for a dead cell dye (Figs. 3G and 3H). We were similarly able to recapitulate the loss of LTi-like ILC3 following IL-2C treatment using alternative gating strategies (Supplementary Figs. S5H and S5I), and could further discount potential loss of canonical surface markers (CCR6, CD127 and c-kit) as an explanation for reduced LTi-like ILC3 (Supplementary Figs. S5J–O).

To determine whether loss of LTi-like ILC3 following IL-2C could be due to extrinsic factors mediated by adaptive immunity—such as Treg expansion or induction of apoptosis via Fas ligation or tumor necrosis factor (TNF)-α—we similarly treated Rag1^−/−^ animals with IL-2C (Supplementary Fig. S6). IL-2C treatment of mice lacking an adaptive immune system similarly induced proliferation in all ILC3 subsets, including LTi-like ILC3, to a comparable extent to wild-type animals (Supplementary Fig. S6A). Moreover, IL-2C treatment led to a reduction of LTi-like ILC3 both as a proportion of total ILC3 and total CD45^+^ cells in Rag1^−/−^ mice (Supplementary Figs. S6B–D). Thus, IL-2C-driven induction of proliferation led to loss of LTi-like ILC3 even in the absence of adaptive immunity. Together these data provoke the hypothesis that in contrast to other ILC subsets LTi-like ILC3 may maintain a quiescent state to facilitate survival in the intestinal tract.

### Bcl-2 supports LTi-like ILC3 survival

**Fig. 4:**
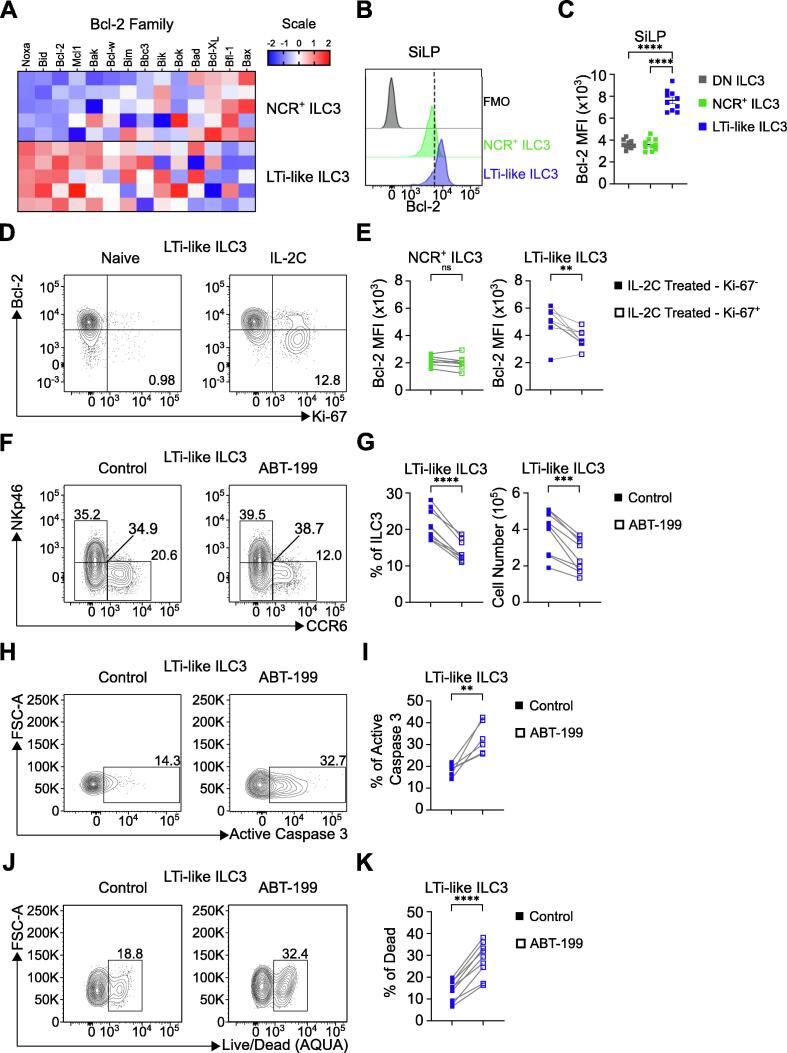
Bcl-2 is a key regulator of LTi-like ILC3 survival. (A) Heatmap showing the relative expression levels of Bcl-2 family members determined via bulk RNA-sequencing of NCR^+^ ILC3 and LTi-like ILC3 from SiLP. (B–C) Bcl-2 expression of NCR^+^ ILC3 and LTi-like ILC3 in SiLP. (D) Bcl-2 and Ki-67 expression in LTi-like ILC3 in SiLP from naïve or IL-2C treated mice. (E) Bcl-2 MFI in Ki-67^−^ and Ki-67^+^ populations of NCR^+^ ILC3 and LTi-like ILC3 in SiLP from IL-2C treated mice. (F–G) Frequency and cell counts of LTi-like ILC3 in SiLP ± ABT-199 treatment *ex vivo*. (H–K) Frequency of active caspase 3 expression (H–I) and live/dead dye acquisition (J–K) in LTi-like ILC3 in SiLP ± ABT-199 treatment *ex vivo*. RNA-sequencing data (A) shown as Z-scores relative to overall average. ILC3 subsets in panels B–G identified via gating strategy in Supplementary Fig. S1A, panels H–K as in Supplementary Fig. S1B. Data shown as mean ± standard error of mean (C) or individual data points (E, G, I, K) and represent two (I) or three independent experiments (C, E, G and K) (*n* = 5–10). Numbers in flow plot indicate percentage of cells in the respective gate. Data in panels E, G, I and K represent paired samples from same animals with or without treatment. * *p* < 0.05, ** *p* < 0.01, *** *p* < 0.001, **** *p* < 0.0001 using unpaired t test (C) or paired t test (E, G, I, K). Bcl-2 = B cell lymphoma 2; DN = double negative; ILC3 = group 3 innate lymphoid cell; IL-2C = interleukin-2 complex; LTi-like = lymphoid tissue-inducer-like; MFI = mean fluorescence intensity; ns = not significant; NCR = natural cytotoxicity receptor-expressing.

To determine the underpinning mechanisms through which quiescent LTi-like ILC3 are maintained within tissues, we first generated bulk RNA-sequencing data of wild-type SiLP NCR^+^ ILC3s and LTi-like ILC3s to identify candidate pathways, which revealed a differential relative expression of pro-survival and pro-apoptotic factor gene families (Fig. 4A). Specifically, LTi-like ILC3s were associated with higher expression of canonical anti-apoptotic genes of the Bcl2 family when compared to NCR^+^ ILC3, including *Bcl2*, *Mcl1* and *Bcl-w* (Fig. 4A). In line with this signature, we could confirm higher expression of Bcl-2 protein in LTi-like ILC3 across multiple tissues when compared to NCR^+^ ILC3s and ILC2 via flow cytometry (Figs. 4B and 4C; Supplementary Figs. S7A–D). As we had previously observed that IL-2C-driven induction of proliferation led to cell loss, we next determined whether this was associated with changes in Bcl-2 expression. Strikingly, we observed LTi-like ILC3 expressing Ki-67 exhibited a significantly reduced expression of Bcl-2 following IL-2C treatment, suggesting Bcl-2 is associated with a non-proliferative state in LTi-like ILC3 (Figs. 4D and 4E). In contrast infection with *C. rodentium*, which was not associated with proliferation in LTi-like ILC3 (Fig. 2), did not change expression of Bcl2—which remained at levels comparable with naïve mice (Supplementary Figs. S7E and S7F). Notably, NCR^+^ ILC3 expressed higher levels of the pro-survival protein Bcl-xL than LTi-like ILC3 at steady state (Supplementary Figs. S7G and S7H). However, upon IL-2C treatment proliferating LTi-like ILC3 also exhibited moderate increases in expression of Bcl-xL (Supplementary Figs. S7I and S7J), indicating further changes to Bcl-2 family proteins in proliferating ILC3.

To definitively test the importance of Bcl-2 expression in LTi-like ILC3 survival we performed an *ex vivo* culture of SiLP cells with the selective Bcl-2 inhibitor ABT-199. Within 4 hours of incubation with ABT-199 *ex vivo* we observed a clear and selective reduction in SiLP LTi-like ILC3 in both frequency and cell number (Figs. 4F and 4G), while in contrast DN ILC3 and NCR^+^ ILC3 survival was unaffected (Supplementary Figs. S7K and S7L). A similar loss of LTi-like ILC3 was also observed following *ex vivo* culture of mesenteric lymph nodes cells with ABT-199 (Supplementary Figs. S7M and S7N). Loss of LTi-like ILC3 could not be explained by downregulation of surface CCR6, and we failed to observe the emergence of a compensatory CCR6^−^ population among T-bet- ILC3 following ABT-199 treatment (Supplementary Figs. S7O and S7P). To determine whether inhibition of Bcl-2 was inducing cell death in LTi-like ILC3, we measured the expression of active caspase 3 and uptake of a fixable viability dye. Consistent with the reduction in LTi-like ILC3, we saw elevated expression of active caspase 3 and significantly greater acquisition of dead cell dye within LTi-like ILC3 compared to NCR^+^ ILC3s (Figs. 4H–K; Supplementary Figs. S7Q–S), further implicating Bcl-2 activity as critical for LTi-like ILC3 survival. Together, these findings identify Bcl-2 as a key pro-survival molecule for quiescent LTi-like ILC3s.

### Bcl-2 protects against OXPHOS-mediated metabolic stress in LTi-like ILC3

**Fig. 5:**
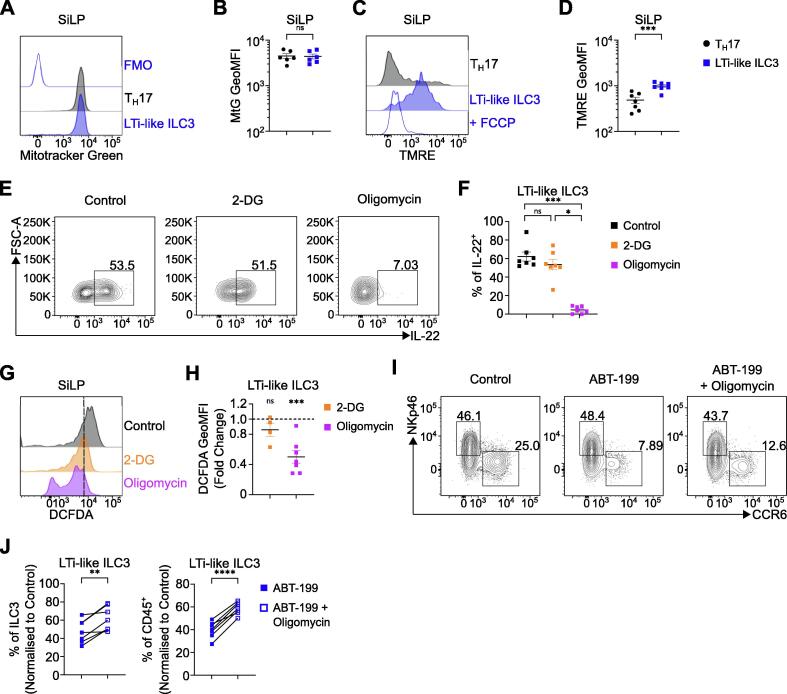
Bcl-2 protects against oxidative phosphorylation-mediated metabolic stress. (A–B) Representative histogram of mitochondrial mass and MFI in T_H_17 cells and LTi-like ILC3 in SiLP. (C–D) Representative histogram of mitochondrial activity/polarization and MFI in T_H_17 cells and LTi-like ILC3 in SiLP. (E–F) IL-22 production of *ex vivo* IL-1β and IL-23 stimulated SiLP LTi-like ILC3 ± 2-DG or Oligomycin treatment. (G–H) Representative histogram of cellular ROS (DCFDA) and DCFDA MFI fold change relative to control following 2-DG or Oligomycin treatment *ex vivo* in LTi-like ILC3 in SiLP. (I–J) Frequency of LTi-like ILC3 ± ABT-199 and Oligomycin treatment *ex vivo* in SiLP. T_H_17 cell and ILC3 subsets in panels A–D, and G–H identified via gating strategy in Supplementary Fig. S1B, panels E–F and I–J as described in Supplementary Fig. S1A. Data shown as mean ± standard error of mean (B, D, F, H) or individual data points (J) and represent two (F, 2-DG in H) or three independent experiments (B, D, H, J) (*n* = 4–7). Numbers in flow plot indicate percentage of cells in the respective gate. Data in panel J represents paired samples from same animals with or without treatment. * *p* < 0.05, ** *p* < 0.01, *** *p* < 0.001, **** *p* < 0.0001 using unpaired t test (B, D, H), paired t test (J) or Dunn's multiple comparisons test (F). 2-DG = 2-deoxyglucose; Bcl-2 = B cell lymphoma 2; CD = clusters of differentiation; DCFDA = 2′,7′-dichlorofluorescein diacetate; ILC3 = group 3 innate lymphoid cell; IL = interleukin; LTi-like = lymphoid tissue-inducer-like; MFI = geometric mean fluorescence intensity; ns = not significant; T_H_17 = T helper 17; TMRE = Tetramethylrhodamine, ethyl ester.

Our data suggest LTi-like ILC3 readily performs effector functions while maintaining a quiescent-like state. These findings raise the question of how the demands of effector function and long-term persistence are met without cellular proliferation and population expansion. Recent studies have highlighted a key role for metabolism in supporting LTi-like ILC3 effector function[33], [34], [35]. Thus, we first analyzed mitochondrial content and activity and noted that while LTi-like ILC3 had comparable levels of mitochondrial mass to analogous Th17 cells (Figs. 5A–B), ILC subsets—including LTi-like ILC3—exhibited significantly greater mitochondrial potential (Figs. 5C–D; Supplementary Figs. S8A–D). To determine whether mitochondrial metabolism helped to support effector function in LTi-like ILC3, we cultured cells with inhibitors of glycolysis (2-deoxyglucose; 2-DG) and OXPHOS (oligomycin). Notably oligomycin treatment, but not 2-DG treatment, resulted in a marked reduction of IL-22 production by LTi-like ILC3 (Figs. 5E–F), as well as other ILC3 subsets (Supplementary Fig. S8E), indicating OXPHOS is utilized by ILC3 to support optimal effector functions. Mitochondrial potential remained elevated—although unchanged—in colonic LTi-like ILC3 in response to *C. rodentium* infection (Supplementary Figs. S8F and S8G), and infection-induced IL-22 increases remained sensitive to oligomycin-mediated ablation (Supplementary Figs. S8H and S8I). Importantly, oligomycin treatment alone was not sufficient to alter frequencies of LTi-like ILC3 or other ILC family members (Supplementary Figs. S8J and S8K), suggesting oligomycin-mediated metabolic inhibition, and not cell death, prevents ILC3 effector production. We next hypothesized that stress associated with the metabolic activity required to fuel IL-22 production may be in part offset by pro-survival factors. Indeed Bcl-2 family members can mitigate mitochondrial metabolic stress associated with the production of reactive oxygen species (ROS) to prevent apoptosis^36^. In line with this, we detected high levels of cellular ROS in LTi-like ILC3, assessed via 2′,7′-dichlorofluorescein diacetate (DCFDA) assay, that were reduced following incubation with oligomycin, but not 2-DG (Figs. 5G and 5H; Supplementary Fig. S8L). To determine whether Bcl-2 acts to facilitate LTi-like ILC3 persistence by protecting against metabolic stress, we cultured LTi-like ILC3 *ex vivo* with ABT-199 in the presence or absence of oligomycin. Notably, inhibition of OXPHOS concurrent with inhibition of Bcl-2 could partly rescue cell death in LTi-like ILC3 (Figs. 5I and 5J), but in contrast did not increase frequencies of NCR^+^ ILC3 (Supplementary Fig. S8M), suggesting that the metabolic stress from mitochondrial respiration required to maintain effector functions in LTi-like ILC3 is in part negated by expression of Bcl-2 in order to prevent apoptosis (Supplementary Fig. S8N).

### Discussion

Here, we show that LTi-like ILC3 in the gastrointestinal tract are maintained largely independently of hematopoietic replenishment at steady state through a pro-survival program that negates the metabolic stress associated with facilitating effector function in a quiescent state. Moreover, our data further highlight the differential biology between LTi-like ILC3 and other ILC subsets, including DN and NCR^+^ ILC3. Strikingly, we demonstrate that LTi-like ILC3 are typically non-proliferative cells, both at steady state and during a robust effector cytokine response to *C. rodentium* infection, and instead are associated with high levels of the pro-survival molecule Bcl-2 that protects against stress associated cell death.

The ontogeny of LTi-like ILC3 has until recently been unclear. Fetal LTi that arise during embryogenesis were previously shown to persist, but also to be partially replaced immediately after birth^12^. However, whether hematopoietic input continues to replenish ILC3 subsets beyond weaning—once populations have been fully established—has been unclear. Here we demonstrate relatively low levels of replenishment of LTi-like ILC3 during adulthood and persistence of mature tissue-resident ILC3 for periods of 10 weeks and over—in contrast with early reports estimating the half-life of LTi-like ILC3 at 22-26 days^14^. One possible explanation for the discrepancy between these estimates could be the life stage in which ILC3 turnover was determined. For example, Sawa *et al* determined turnover of ILC3 during the initial neonatal period of seeding and weaning, where a final wave of hematopoietic seeding of ILC has recently been reported to occur^12^. In contrast, here we focus on the replenishment of established ILC3 populations in adult intestinal tissue. Adding to these prior studies, our findings suggest that while hematopoiesis may contribute to the initial colonization of intestinal tissues by ILC3 subsets, this is likely limited to early life—with only negligible turnover and hematopoietic contribution beyond weaning and in the adult gut.

Our data further suggest LTi-like ILC3 are a quiescent-like population and unlikely to be maintained through homeostatic proliferation and self-renewal. Surprisingly, LTi-like ILC3 were found to be refractory to proliferation even during infection with *C. rodentium*. Moreover, although IL-2C treatment could induce proliferation of LTi-like ILC3 it also led to a specific loss of LTi-like ILC3. The reasons for this latter observation are not yet fully clear, however, the correlation with Bcl-2 downregulation could in part explain why proliferation leads to loss of LTi-like ILC3 *in vivo.* This biology is reminiscent of previous findings demonstrating high expression of Bcl-2 in non-cycling NK cells which is subsequently lost upon entry into cell cycle^37^. Moreover, the identification of a strong pro-survival program in LTi-like ILC3 characterized by high levels of Bcl-2 is in line with previous observations^38^, and intriguingly similar to that described for other quiescent intestinal immune cell populations such as tissue-resident memory T cells^39^.

The transcriptional or environmental cues that imprint and maintain a Bcl-2-associated and quiescent-like phenotype on LTi-like ILC3 remain unclear. In contrast to NCR^+^ ILC3 which patrol the lamina propria^21^, LTi-like ILC3 are typically restricted to lymphoid structures in healthy animals. There they co-localize with stromal cells that provide survival cues such as IL-7 and stem cell factor^40^, while also being subject to dietary, microbial and neuronal input^41^. Prior studies have demonstrated that inflammatory cues or genetic-induced deletion of niche-retaining chemokine receptor expression drive the mobilization of LTi-like ILC3 away from organized lymphoid structures, such as cryptopatches, and into the villi where they become proliferative[17], [42], [43]. These findings implicate the tissue niche as a potential determinant of the proliferative capacity, long-term survival and persistence of LTi-like ILC3, although the precise cues that confer a quiescent and anti-apoptotic program remain to be determined. Therefore, future studies are required to determine if quiescence and persistence of LTi-like ILC3 in the gut are directly dependent upon the cellular niche and tissue microenvironment in which they are localized.

Together our findings suggest LTi-like ILC3 are a quiescent long-lived cell with minimal turnover at steady state. Prior studies demonstrated an intestinal resident RORγt^+^ T-bet^+^ DN ILC3 population lacking expression of mature ILC3 subset surface markers (e.g. CCR6 and NKp46) acts as an immature pool of precursors for NCR^+^ ILC3, but demonstrated no capacity to transdifferentiate into LTi-like ILC3^23^. In line with their transcriptional and functional overlap with NCR^+^ ILC3, DN ILC3 were also found to be proliferative and expressed lower levels of Bcl-2 than LTi-like ILC3. Our data further emphasize the developmental, phenotypic and functional distinction between LTi-like ILC3 and the T-bet co-expressing ILC3 lineage^15^. Prior evidence has demonstrated LTi precursors to diverge from the remaining ILC lineage earlier during development^44^, and conversely LTi-like ILC3 progeny cannot be generated from the common helper ILC progenitor—which conversely gives rise to DN and NCR^+^ ILC3^45^. Thus, while we cannot completely rule out the contribution of an as-yet unidentified tissue-resident progenitor to LTi-like ILC3 maintenance, our data—together with current understanding—would suggest cell-intrinsic survival mechanisms favor long-term persistence of LTi-like ILC3 without significant input from peripheral progenitors.

Finally, we highlight ILC-intrinsic metabolism as a critical determinant of effector function and cell fate, in line with a number of recent studies[33], [34], [46]. Here we demonstrate mitochondrial metabolism is required to support effector cytokine production and that, in the absence of Bcl-2-mediated protection, metabolic stress may lead to cell death *ex vivo*. In contrast, IL-2-driven proliferation resulted in a reduction in LTi-like ILC3 and intrinsic Bcl-2 expression. A previous study demonstrated IL-2 induction of ILC3 proliferation requires mTOR^35^, suggesting such signals may remodel the metabolic program of ILC3 to facilitate these demands^34^. One possibility is that the metabolic stress associated with proliferation may initially be detrimental to LTi-like ILC3 survival, but that a small proportion of cells that survive this initial stimulation may undergo significant metabolic reprogramming. Indeed, while overall LTi-like ILC3 frequencies and numbers were reduced following treatment with IL-2C, and associated with loss of Bcl-2 expression, we observed upregulation of the pro-survival molecule Bcl-xL in the remaining proliferating Bcl-2^lo^ LTi-like ILC3—suggesting a small number of proliferative LTi-like ILC3 may persist. Intriguingly, one recent report suggested metabolic reprogramming of ILC3 following an initial activation facilitates a trained proliferative response to repeat challenges^33^. Nonetheless, a limitation of this current study was that the investigation of Bcl2 dependency and the metabolic requirements for LTi-like ILC3 survival was restricted to *ex vivo* cultures and the use of inhibitors. Future studies should dissect the cell-intrinsic pathways that determine LTi-like ILC3 quiescence, effector function and survival *in vivo* via conditional knockout approaches, and further consider the nutritional context and survival cues present within LTi-like ILC3-rich microenvironments.

Taken together our data further emphasize differential biology of intestinal ILC3 subsets and advance our understanding the molecular mechanisms that underpin their survival, persistence, and effector functions. Further studies to uncover the interplay between tissue niche, cell-intrinsic metabolism and ILC3 subset dynamics may help to uncover the contributions of these cells to tissue health or chronic inflammatory diseases in the gastrointestinal tract.

## METHODS

### Mice

Age- and sex-matched mice between 8–12 weeks of age were used. *Cxcr4*^CreERT2^ ROSA^tdTomato^ (ROSA^tdT^)^27^ and *Id2*^CreERT2^ ROSA^tdRFP25,26^ mouse models have been previously described. *Cxcr4*^CreERT2^ ROSA^tdT^ were maintained at the Queens Medical Research Institute (Edinburgh, UK). Rag1^−/−^ mice and C57BL/6 mice were bred and maintained at the University of Manchester or purchased from Envigo laboratories (C57BL/6). All mice were maintained under specific pathogen-free conditions and provided with food and water ad libitum.

### Tamoxifen treatment

ID2-^CreERT2^ ROSA^tdRFP^ reporter model, 8-week-old mice received 5 doses of 5 mg tamoxifen (Sigma-Aldrich) diluted in 5 μl of ethanol and 95 μl of corn oil (Sigma-Aldrich) via oral gavage on days 0, 2, 4, 7, and 9. Mice were culled either 10 days or 52 days post-final gavage. CXCR4-^CreERT2^ ROSA^tdT^ reporter model, 6–12-week-old mice received 3 doses of 0.12 mg tamoxifen per gram of body weight for 3 consecutive days in a 100 μl bolus. Mice were culled 12 weeks post-final gavage.

### IL-2 complex treatment

IL-2C was made by mixing 2.5 ug of IL-2 monoclonal antibody (JES6-1A12, BioXCell) with 7.5 ug recombinant IL-2 (ThermoFisher) in 200 μl of phosphate-buffered saline (PBS) for 20 minutes at 37°C. Three doses of IL-2C were injected via intraperitoneal over 6 days and mice were sacrificed 2 days after the last dose.

### Bone marrow chimeras

For intestinal shielded bone marrow chimera, recipients were anesthetized with ketamine (80 mg/kg; Vetoquinol) and xylazine (8 mg/kg; Bayer) via intraperitoneal injection. CD45.2^+^ C57BL/6 mice (Envigo laboratories), were selectively irradiated by placing a lead shield over the abdomen and irradiated with two doses of 5.5 grays of radiation, 5 minutes per dose. Recipient mice were reconstituted with 5 x 10^6^ CD45.1^+^ congenic bone marrow cells via intravenous infusion. Mice were analyzed 10–20 weeks later as indicated. For whole-body chimeras, CD45.2^+^ C57BL/6 mice (Envigo laboratories) were irradiated using the same approach without the lead shield. Mice were then reconstituted with 50/50 CD45.1^+^/CD45.2^+^ bone marrow via intravenous infusion and analyzed 32 weeks later. Mice received 0.03% enrofloxacin (Bayer) in the drinking water 1 week before and for the first 4 weeks after irradiation and were housed with sterile water, diet, and bedding.

### *C. rodentium* culture and infection

*C. rodentium* (nalidixic acid resistant strain) was originally a kind gift from Gad Frankel (Imperial College London). *C. rodentium* was cultured from glycerol stock in 10 ml of Luria-Bertani (LB; Merck) broth containing nalidixic acid (50 μg/ml) for 12–18 hours at 37°C, shaking at 200 rpm. The resulting bacteria was then streaked onto an LB agar (Sigma-Aldrich) plate containing nalidixic acid (Sigma-Aldrich) and left at 37°C for 24 hours. Singles colonies were picked and grown in 10 ml of LB broth containing nalidixic acid for 12–18 hours. Bacteria were then centrifuged at 3000 xg for 10 minutes and resuspended in 1 ml of sterile PBS. Infective doses (∼ 2 x 10^9^ colony forming unit) of *C. rodentium* were confirmed via plating and administered to mice via oral gavage.

### Tissue digestion

Intestinal lamina propria lymphocytes were isolated from intestinal tissue by removing fat and Peyer’s patches and opened longitudinally. Luminal contents were removed by vigorous vortexing in PBS and then the epithelial lining was removed by repeat incubation in PBS containing 1 mM Ethylenediaminetetraacetic acid  (EDTA; Invitrogen), 1 mM dithiothreitol (DTT; Sigma-Aldrich) and 5% fetal bovine serum (FBS; Gibco-Life Technologies) for 15 minutes with constant agitation at 37°C. Tissue was then digested in 10 ml of complete media (10% FBS, 100 units/ml penicillin, 100 μg/ml streptomycin, 2 mM L-glutamine in (RPMI media)(all Sigma-Aldrich) containing 20 μg/ml DNase I (Sigma-Aldrich) and either for Collagenase VIII (Sigma-Aldrich) for 25 minutes, or Collagenase/Dispase for 45 minutes, vortexing at 37°C. Collagenase Dispase (Roche) was used for experiments where phenotyping of ILC3 was performed while Collagenase VIII was used to increase cell yields for *ex vivo* assays. Subsequently, digested tissue was filtered through a 100 μM nylon filter and a 70 μM nylon filter. Filtrate was then centrifuged at 500 xg for 5 minutes to pellet lamina propria lymphocytes.

### Flow cytometry

Single-cell preparations were stained with the following antibodies:

**Table:** Flow Cytometry Antibodies

**Surface**		
Anti-mouse B220	Clone RA3-6B2	ThermoFisher
Anti-mouse CCR6	Clone 140706	BD Biosciences
Anti-mouse CD3	Clone 145-2C11 or Clone 17A2	ThermoFisher
Anti-mouse CD4	Clone GK1.5	BD Biosciences
Anti-mouse CD5	Clone 53-7.3	ThermoFisher
Anti-mouse CD11b	Clone M1/70	ThermoFisher
Anti-mouse CD11c	Clone N418	ThermoFisher
Anti-mouse CD19	Clone 6D5	BD Biosciences
Anti-mouse CD45	Clone 30-F11	ThermoFisher
Anti-mouse CD45.1	Clone A20	ThermoFisher
Anti-mouse CD45.2	Clone 104	ThermoFisher
Anti-mouse CD49α	Clone Ha31/8	BD Biosciences
Anti-mouse CD64	Clone X54-5/7.1	Biolegend
Anti-mouse CD90.2	Clone 30-H12	ThermoFisher
Anti-mouse CD117 (c-Kit)	Clone 104D2	ThermoFisher
Anti-mouse CD127 (IL-7Rα)	Clone A7R34	ThermoFisher
Anti-mouse F4/80	Clone BM8	Biolegend
Anti-mouse KLRG1	Clone 2F1	ThermoFisher
Anti-mouse Ly6C	Clone AL-21	BD Biosciences
Anti-mouse Ly6G	Clone 1a8	Biolegend
Anti-mouse NK1.1	Clone PK136	BD Biosciences
Anti-mouse NKp46	Clone 29A14	ThermoFisher
**Intracellular**		
Anti-mouse Bcl-2	Clone BCL/10C4	Clone 30-H12
Anti-mouse IL-22	Clone poly5164	Biolegend
Anti-mouse Ki-67	Clone SolA15	ThermoFisher
Anti-mouse RORγt	Clone B2D	ThermoFisher
Anti-mouse Bcl-xL	Clone 54H6	Cell Signalling

Dead cells were excluded from analysis using the LIVE/DEAD Fixable Aqua Dead Cell Stain Kit (Invitrogen). Cells were gated as in Supplementary Fig. S1.

Staining of intracellular markers was performed by using the Foxp3 Transcription Factor Buffer kit (eBioscience). For analysis of cell cycle status, live single-cell preparations were stained with 10 μM Vybrant^TM^ Dye Cycle^TM^ (ThermoFisher) for 30 minutes at 37°C, 5% CO_2_. For analysis of mitochondrial mass, live single-cell preparations were stained with 100 nM MitoTracker™ Green (ThermoFisher) for 30 minutes in Hanks' Balanced Salt Solution (HBSS; Sigma-Aldrich). For analysis of mitochondrial potential/activity, live single-cell preparations were stained 200 nM of Tetramethylrhodamine, Ethyl Ester, Perchlorate (TMRE; Abcam) for 30 minutes in HBSS; As a negative control, live single-cell preparations were co-treated with 50 μM of carbonyl cyanide 4-(trifluoromethoxy) phenylhydrazone (FCCP; Sigma-Aldrich) for 30 minutes in HBSS. For analysis of cellular ROS, live single-cell preparations were stained with 20 μM 2’,7’–dichlorofluorescin diacetate (DCFDA; Abcam) for 5 minutes in HBSS; As a positive control live single-cell preparations were co-treated with 200 μm of tert-butyl hydrogen peroxide (TBHP; Merck) for 5 minutes in HBSS. All flow cytometry data was acquired on a BD Fortessa and analyzed using FlowJo version 10.

### *Ex vivo* assays

#### *Ex vivo* ILC3 cytokine stimulation

5 x 10^6^ SiLP cells were incubated in 250 μl of complete media containing 20 ng/ml IL-1β and IL-23 (R&D Systems) for 2 hours at 37°C, 5% CO_2_. After 2 hours, cells received 50 ul of complete media containing eBioscience™ Cell Stimulation Cocktail plus protein transport inhibitors (ThermoFisher) (2 μl/ml) on top and incubated for a further 3 hours. To measure the impact of metabolic inhibition on cytokine production, 2-DG (2.5 mM; Merck) or Oligomycin (1 μM; Merck) was added when adding IL-1β/-23. After incubation, cells were centrifuged, and cells were used for antibody staining for flow cytometry.

#### Bcl-2 inhibitor incubation

5 x 10^6^ SiLP cells were incubated in 250 μl complete media containing 200 nM ABT-199 (Stratech) for 4 hours at 37°C, 5% CO_2_. To measure the impact of metabolic inhibition on Bcl-2 mediated survival, Oligomycin (1μM) was added when adding ABT-199. After incubation, cells were stained for flow cytometry. To assess active caspase 3 activity the CaspGLOW^TM^ Fluorescein Active caspase-3 Staining Kit (Invitrogen) was used. Cells were incubated in ABT-199 as stated above but incubated with 2.5 μl of DEVD-FMK (FITC-conjugated caspase 3 inhibitor) in the final hour, followed by antibody staining for flow cytometry.

### RNA-sequencing

RNA was isolated from sort-purified NCR^+^ ILC3 and LTi-like ILC3 from SiLP using an RNA Purification Kit (Norgen) and amplified using SMART-Seq^TM^ v4 Ultra Low Input RNA Kit for sequencing (Takara Bio USA, Inc.), producing double-stranded complementary DNA. Complementary DNA was purified with AMPure XP beads and quantified with Qubit (Life Technologies^TM^). Library preparation was done using NEBNext Ultra RNA Library Prep Kit (New England Biolabs) following manufacturer recommendations. Libraries were then sequenced on Illumina NovaSeq 6000 S4 flowcell with PE150 according to results from library quality control and expected data volume. CASVA-based recognition was used to convert raw counts into FASTQ files. Reads with adapter contamination, >10% uncertain nucleotides, or had a base quality of <5 in over 50% of the read were filtered out. Remaining reads were aligned with HISAT2. Differential expression analysis was performed using R software and DeSeq2 package. Z-scores were calculated from the resulting differential expression output.

### Statistics

All graphs and statistical analysis were carried out in GraphPad Prism 7. Group *n* numbers are stated in each figure legend along with statistical test utilized. Parametric or non-parametric tests were based on Shapiro-Wilk normality test outcome. Mean fluorescence intensities were calculated via FlowJo version 10.6.1.

## AUTHOR CONTRIBUTIONS

J.I.K. – conceptualization, investigation, data curation, formal analysis, methodology, visualization, writing, reviewing, and editing of manuscript. F.M.G., B.M-D., R.T-P., M.S.M, S.H.H. – investigation, data curation, formal analysis. X.RR. – resources, supervision. R.G. – resources, conceptualization, supervision, validation. M.R.H. – conceptualization, investigation, data curation, formal analysis, visualization, funding acquisition, project administration, supervision, writing, reviewing, and editing of manuscript.

## DECLARATIONS OF COMPETING INTEREST

James King and the Hepworth lab declare financial support from AstraZeneca as part of an Medical Research Council (MRC)-funded industrial Cooperative Awards in Science and Technology (CASE) PhD studentship.

## FUNDING

Research in the Gentek Laboratory is supported by a Kennedy Trust for Rheumatology Research Senior Fellowship and a Cancer Research UK Immunology Project Award. Research in the Hepworth Laboratory is supported by a Sir Henry Dale Fellowship jointly funded by the Wellcome Trust and the Royal Society (Grant Number 105644/Z/14/Z), a Biotechnology and Biomedical Sciences Research Council (BBSRC) responsive mode grant (BB/T014482/1) and a Lister Institute of Preventative Medicine Prize.
