# Supplementary material for: Bcl-2 supports survival and metabolic fitness of quiescent tissue-resident ILC3
Source: Mucosal Immunol. 2023 Oct;16(5):658–70. doi: 10.1016/j.mucimm.2023.07.001 (PMC10564625; doi:10.1016/j.mucimm.2023.07.001)
Supplement: Supplementary data 1 [file mmc1.pdf]

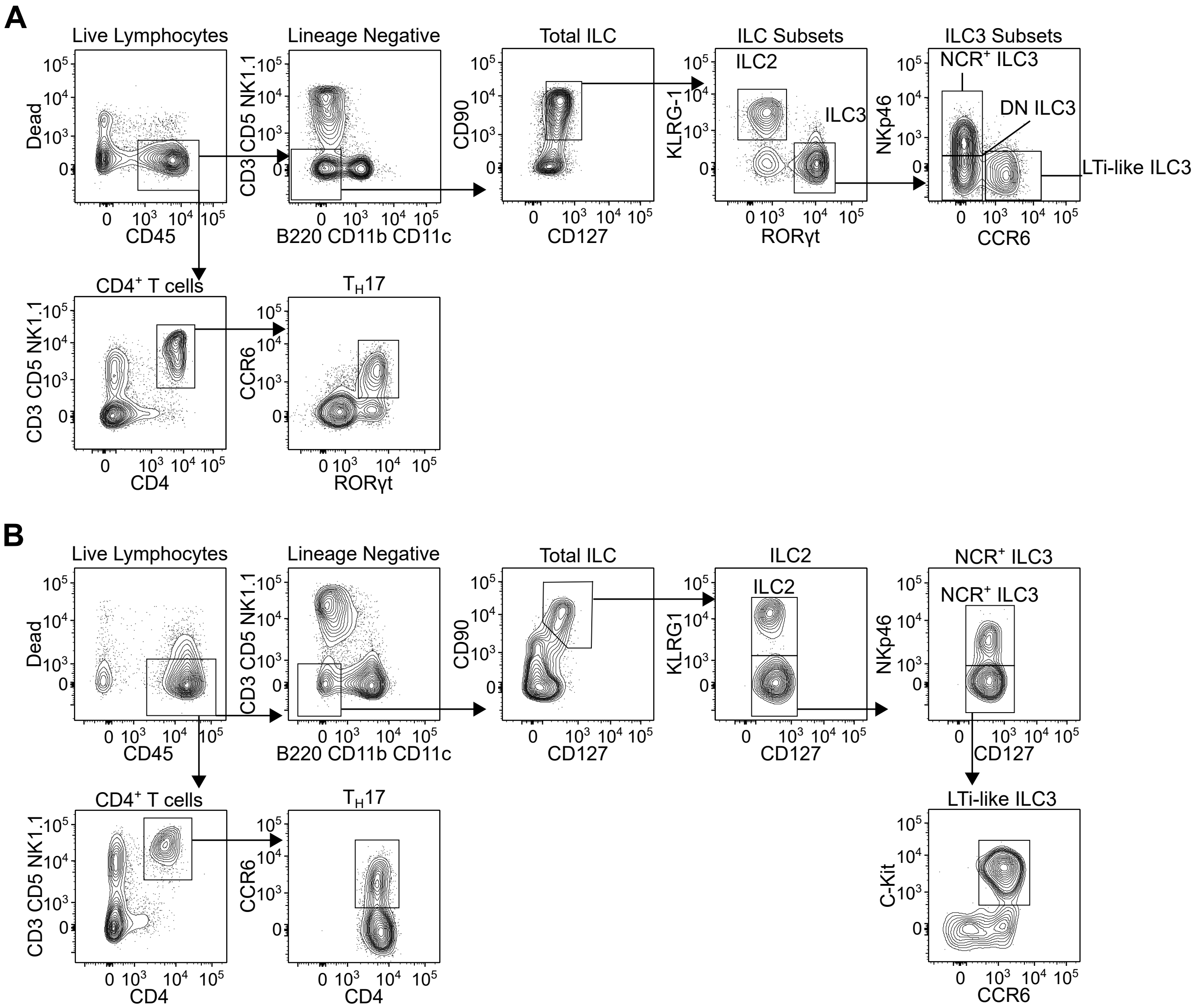

Figure S1. Representative gating strategies to identify ILC3 subsets and Th17 cells. (A) Exemplar gating strategy for intracellular staining; total ILC (Live, $CD45^+$ / Lin^- / $CD90^+$, $CD127^+$) and subsequently ILC2 ($KLRG1^+$, $ROR\gamma t^-$), total ILC3 ($KLRG1^-$, $ROR\gamma t^+$) and ILC3 subsets; DN ILC3 ($CCR6^-$, $NKp46^-$), NCR^+ ILC3 ($CCR6^-$, $NKp46^+$) and LTi-like ILC3 ($CCR6^+$, $NKp46^-$). T_H17 cells were gated as $CD3^+$, $CD5^+$, $NK1.1^+$ / $CD4^+$ / $CCR6^+$, $ROR\gamma t^+$. **(B)** Live cell gating strategy; total ILC as in panel A, ILC2 ($KLRG1^+$), NCR^+ ILC3 ($KLRG1^-$ / $NKp46^+$) and LTi-like ILC3 ($KLRG1^-$ / $NKp46^-$ / $CCR6^+$, $C\text{-}Kit^+$). T_H17 cells were gated as $CD3^+$, $CD5^+$, $NK1.1^+$ / $CD4^+$ / $CCR6^+$.

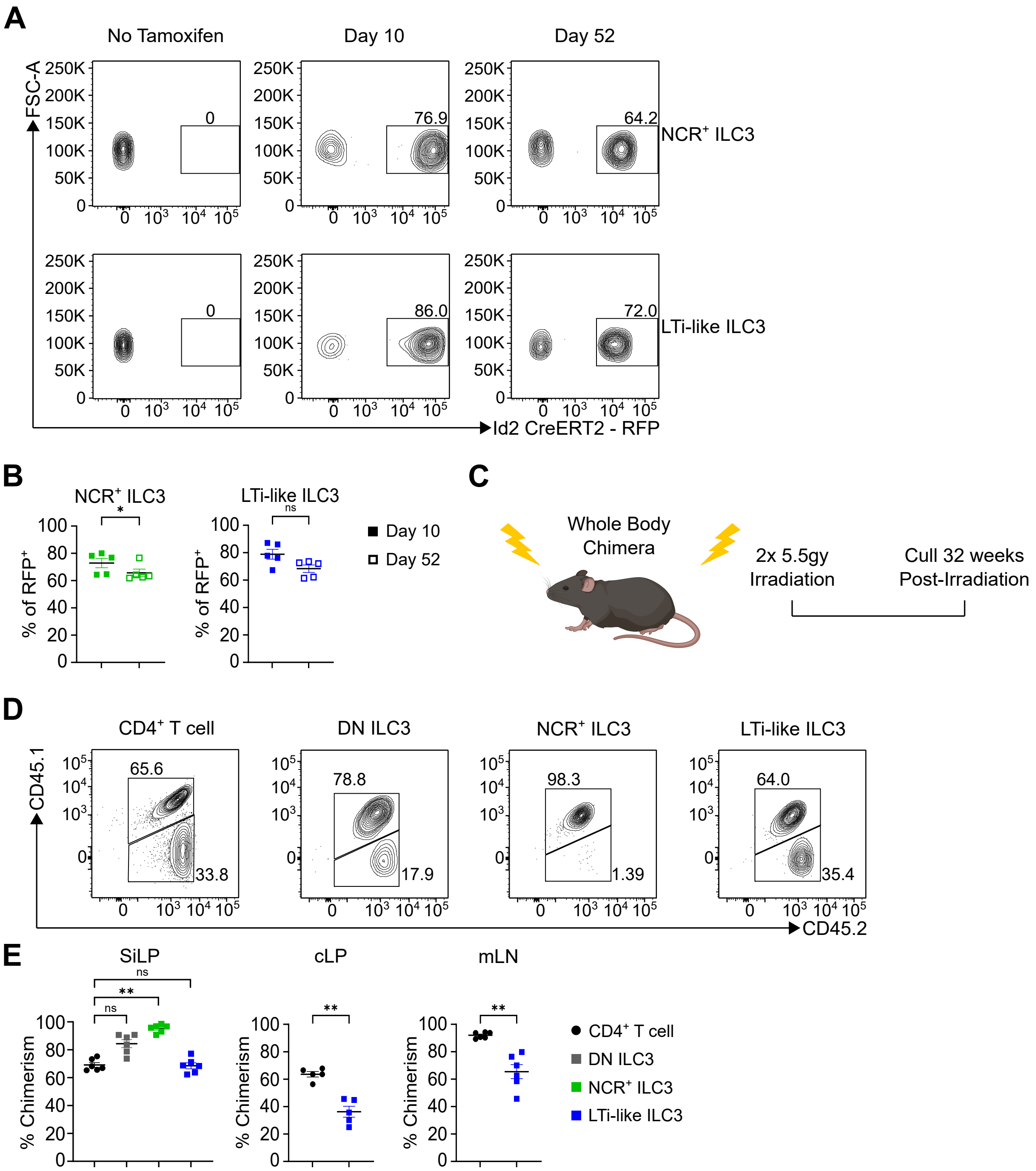

Figure S2. Intestinal ILC3s are maintained independently of bone marrow replenishment. (A-B) Frequency of Id2$^{ERT2Cre-RFP}$ labelled NCR^{+} ILC3s and LTi-like ILC3s in SiLP of no tamoxifen controls, or 10 days or 52 days post-labelling. **(C)** Experimental design for whole body chimeras. Recipient (CD45.2^{+}) adult C57BL/6 mice received sub-lethal whole-body irradiation followed by injection of Donor (CD45.1/2^{+}) bone marrow. Mice were culled 32 weeks post irradiation. **(D-E)** Frequency of CD45.2^{+} CD4^{+} T cells, DN ILC3, NCR^{+} ILC3s, and LTi-like ILC3s in SiLP (D+E), cLP and mLN (E). Panels A-B gated as in Figure S1B, D-E gated as in Figure S1A. Data shown as mean +/- SEM and represent two independent experiments (n = 5 – 6). Numbers in flow plot indicate percentage of cells in the respective gate. *P< 0.05, **P<0.01, ***P<0.001, ****P<0.0001 using Mann-Whitney test (B+E) or Dunn's multiple comparisons test (E; SiLP).

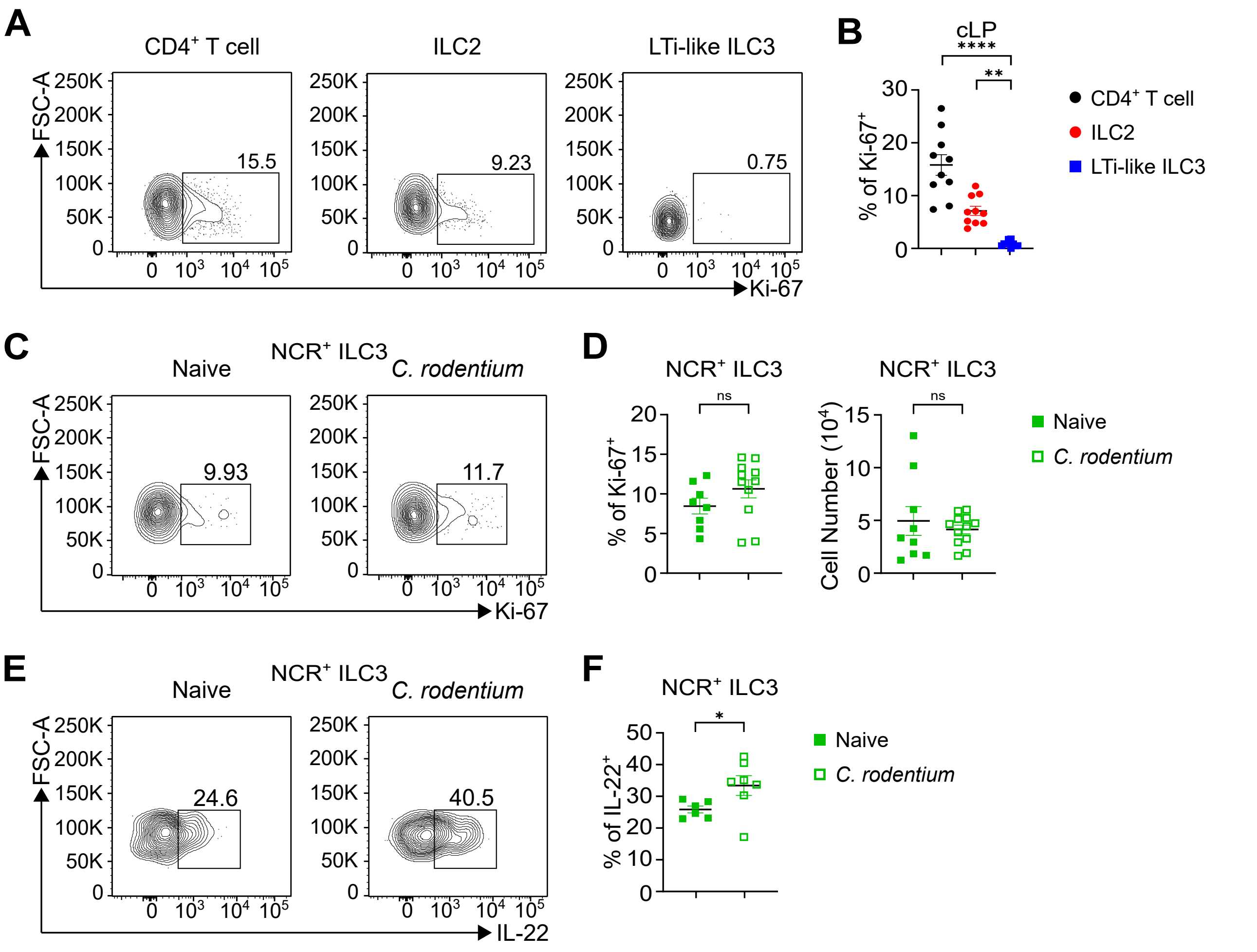

Figure S3. Proliferative capacity of colonic ILC and T cells at steady state and in response to *C. rodentium*. (A-B) Frequency of Ki-67 expression in $CD4^+$ T cell, ILC2 and LTi-like ILC3 in cLP. **(C-D)** Frequency of Ki-67 expression and cell number of NCR^+ ILC3 in cLP of naïve or C. rodentium infected (Day 6) mice. **(E-F)** Frequency of IL-22 production in NCR^+ ILC3 in cLP of naïve or C. rodentium infected (Day 6) mice. $CD4^+$ T cell, ILC2 and ILC3 subsets gated as in Figure S1A. Data shown as mean +/- SEM (n = 9 – 12) and represent three independent experiments. Numbers in flow plot indicate percentage of cells in the respective gate. *P< 0.05, **P<0.01, ***P<0.001, ****P<0.0001 using Holm-Šídák's multiple comparisons test (B), unpaired t-test (D) or Mann-Whitney test (F).

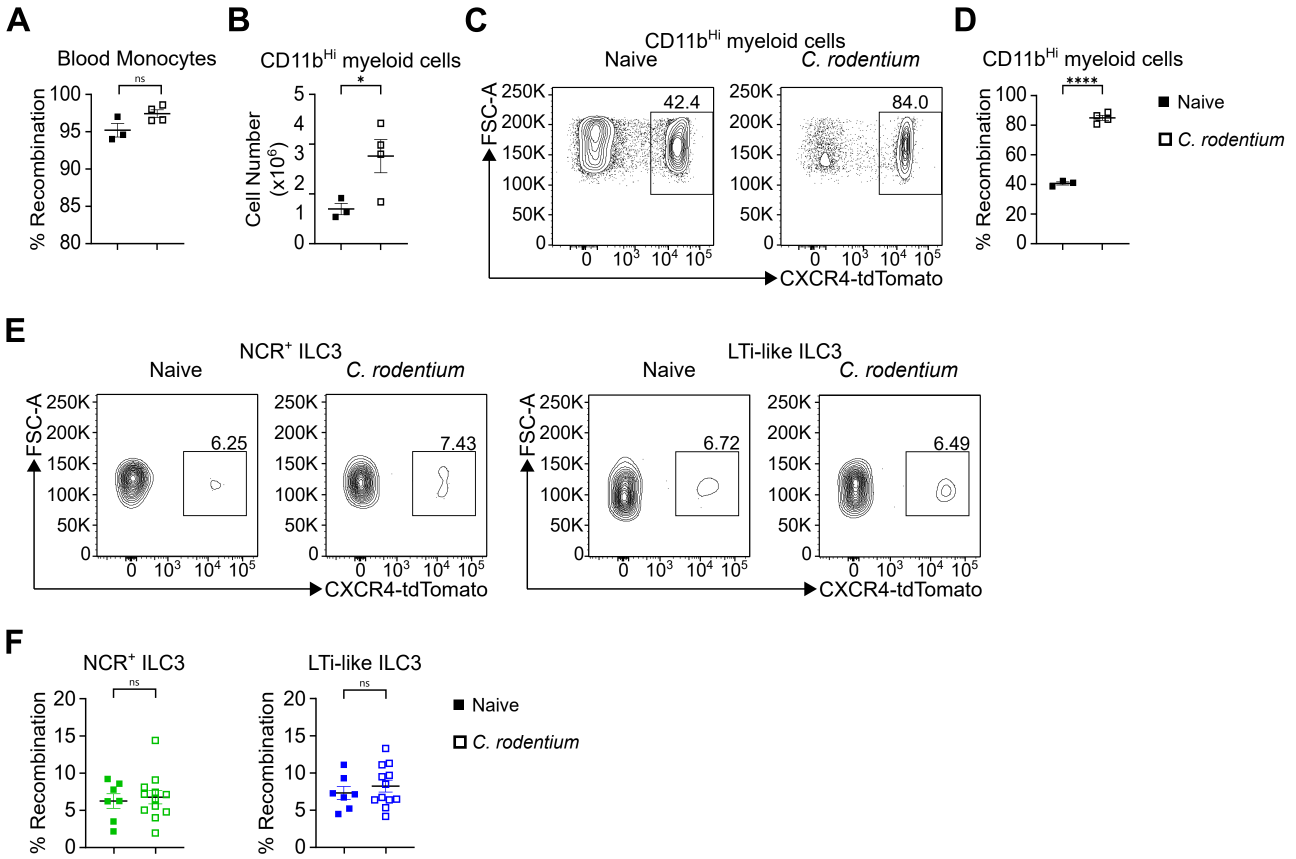

Figure S4. Bone marrow replenishment of ILC3 is negligible during C. rodentium infection. (A) Frequency of tdTomato+ blood monocytes (live CD45^{+} CD11b^{+} Lyc6C^{HI}) in naïve and C. rodentium infected (Day 14) Cxcr4-CreERT2 tdTomato mice. **(B)**, Number of CD11b^{Hi} myeloid cells in colon lamina propria (cLP) of naïve and C. rodentium infected (Day 14) mice. **(C-D)**, Frequency of Cxcr4-CreERT2-tdTomato labelled cLP CD11b^{Hi} myeloid cells in naïve and C. rodentium infected (Day 14) mice. **(E-F),** Frequency of Cxcr4-CreERT2-tdTomato labelled cLP ILC3 subsets in naïve and C. rodentium infected (Day 14) mice. Panels E-F gated as in Figure S1B. Data shown as mean +/- SEM and represent 2 independent experiments (n = 7 - 12). Numbers in flow plot indicate percentage of cells in the respective gate. *P< 0.05, **P<0.01, ***P<0.001, ****P<0.0001 using Unpaired t-test (A, B, F) or Mann Whitney test (D).

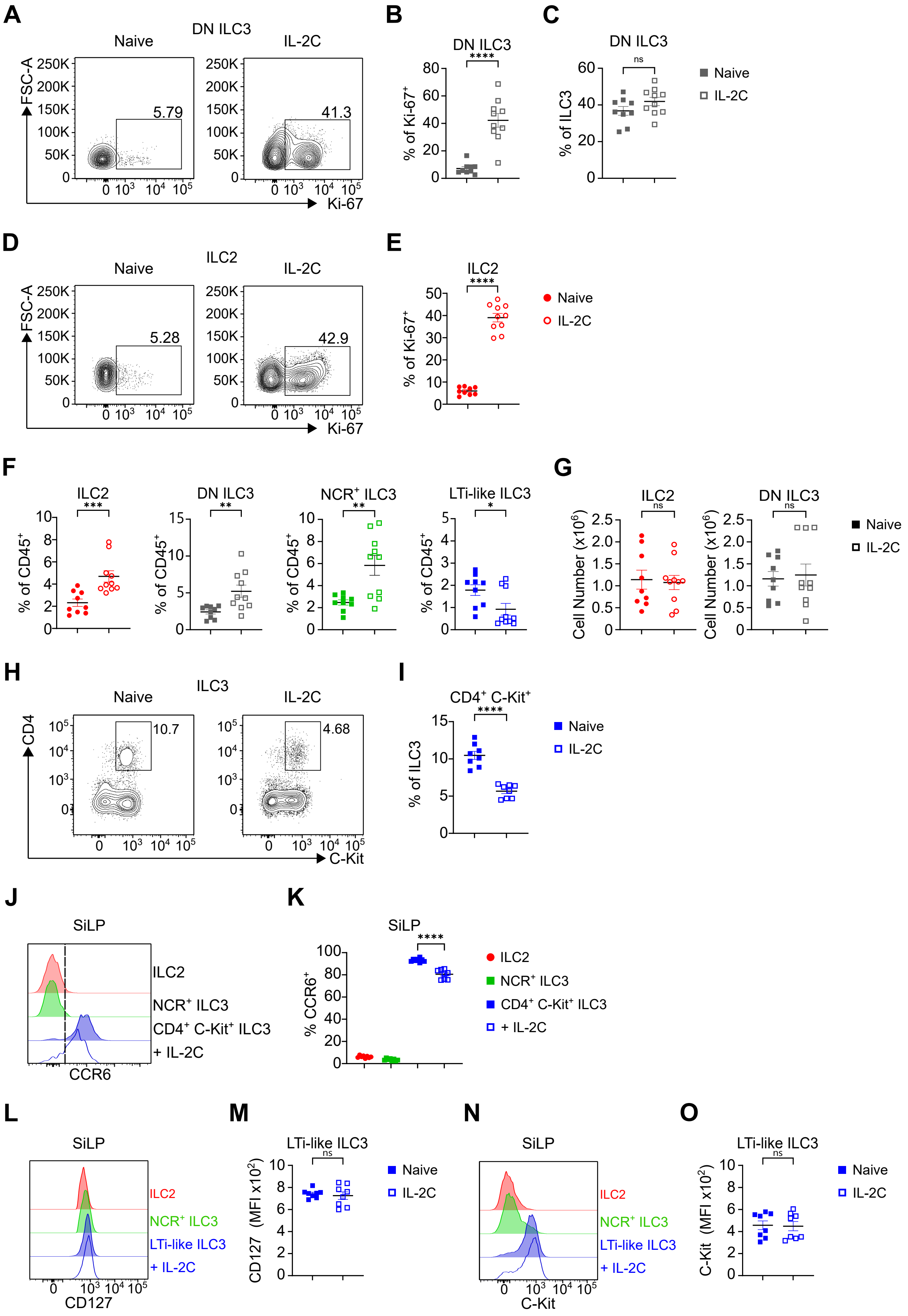

A
DN ILC3
Naive
IL-2C
FSC-A
Ki-67
5.79
41.3
B
DN ILC3
% of Ki-67+
****
C
DN ILC3
% of ILC3
ns
Naive
IL-2C
D
ILC2
Naive
IL-2C
FSC-A
Ki-67
5.28
42.9
E
ILC2
% of Ki-67+
****
Naive
IL-2C
F
ILC2
***
DN ILC3
**
NCR+ ILC3
**
LTi-like ILC3
*
% of CD45+
G
ILC2
ns
DN ILC3
ns
Cell Number (x10^6)
Naive
IL-2C
H
ILC3
Naive
IL-2C
CD4
C-Kit
10.7
4.68
I
CD4+ C-Kit+
% of ILC3
****
Naive
IL-2C
J
SiLP
ILC2
NCR+ ILC3
CD4+ C-Kit+ ILC3
+ IL-2C
CCR6
K
SiLP
% CCR6+
****
ILC2
NCR+ ILC3
CD4+ C-Kit+ ILC3
+ IL-2C
L
SiLP
ILC2
NCR+ ILC3
LTi-like ILC3
+ IL-2C
CD127
M
LTi-like ILC3
ns
CD127 (MFI x10^2)
Naive
IL-2C
N
SiLP
ILC2
NCR+ ILC3
LTi-like ILC3
+ IL-2C
C-Kit
O
LTi-like ILC3
ns
C-Kit (MFI x10^2)
Naive
IL-2C

Figure S5. IL-2C-induces proliferation of ILC but decreases frequency of LTi-like ILC3. **(A-B)** Frequency of Ki-67 expression in siLP DN ILC3 in naïve or IL-2C treated mice. **(C)** Frequency of siLP DN ILC3 in naïve or IL-2C treated mice. **(D-E)** Frequency of Ki-67 expression in ILC2 in SiLP in naïve or IL-2C treated mice. **(F-G)** Frequency as a percentage of $CD45^+$ and cell number of siLP ILC2 and ILC3 subsets in naïve or IL-2C treated mice. ILC2 and ILC3 subsets gated as in Figure S1A. **(H-I)** Representative flow plots and quantification of LTi-like ILC3 percentages in SiLP of IL-2C treated mice using an alternative gating strategy (c-kit^+ $CD4^+$), and **(J-K)** expression of CCR6 on LTi-like ILC3 gating as in H-I compared to other ILC subsets. **(L-O)** representative histograms and MFI quantification of CD127 (L-M) and c-kit (N-O) on LTi-like ILC3 compared to other ILC subsets. Data shown as mean +/- SEM and represent three independent experiments (n = 8 – 12). Numbers in flow plot indicate percentage of cells in the respective gate. *P< 0.05, **P<0.01, ***P<0.001, ****P<0.0001 using Mann-Whitney test.

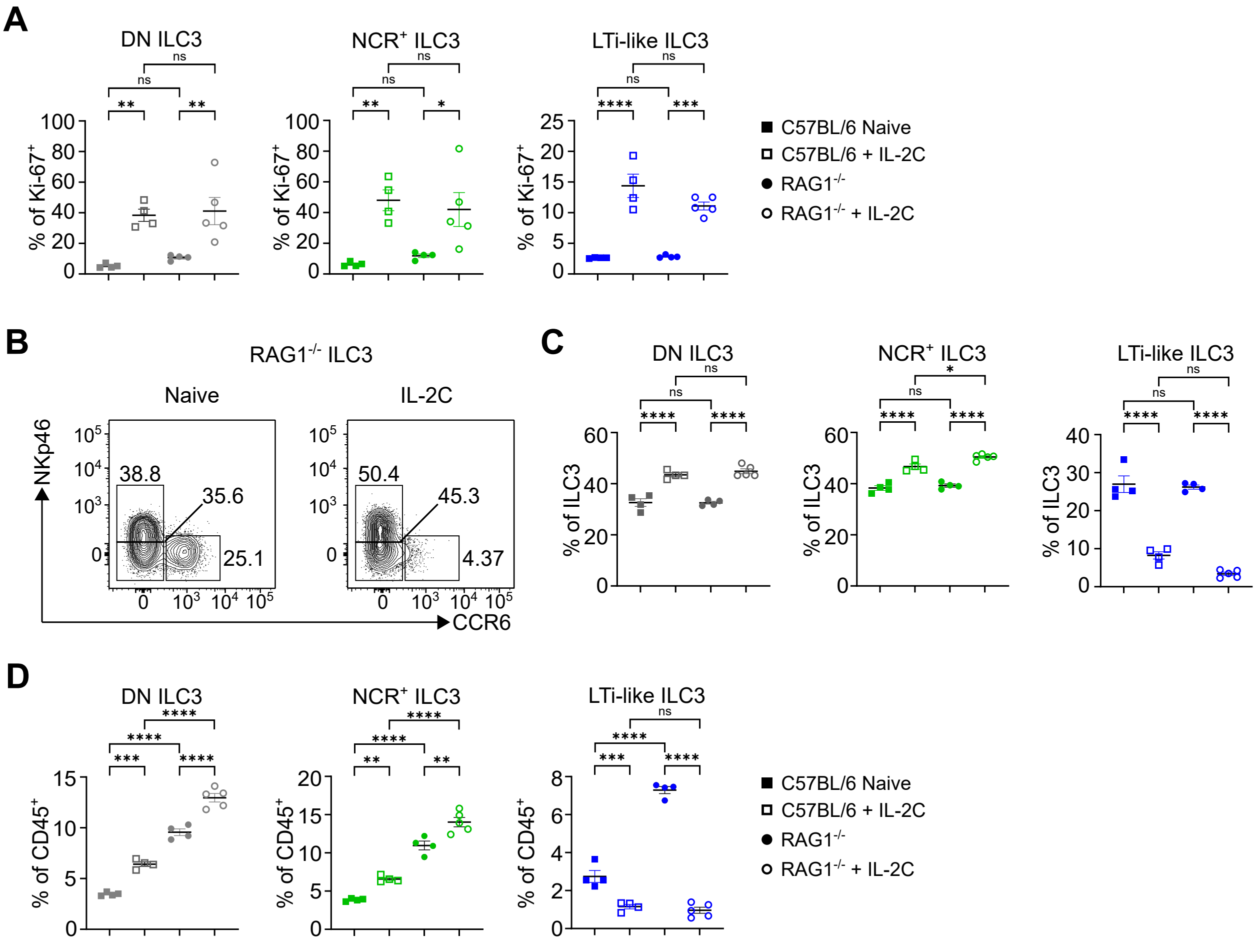

Figure S6. IL-2C-induced loss of LTi-like ILC3 is independent of adaptive immunity. (A) Ki-67 expression in siLP ILC3 subsets in naïve or IL-2C treated C57BL/6 or RAG1$^{-/-}$ mice. **(B-D)** Frequency of siLP ILC3 subsets in naïve or IL-2C treated C57BL/6 or RAG1$^{-/-}$ mice. ILC3 subsets gated as in Figure S1A. Data shown as mean +/- SEM and represent two independent experiments (n = 7-8). Numbers in flow plot indicate percentage of cells in the respective gate. *P< 0.05, **P<0.01, ***P<0.001, ****P<0.0001 using Tukey's multiple comparison test.

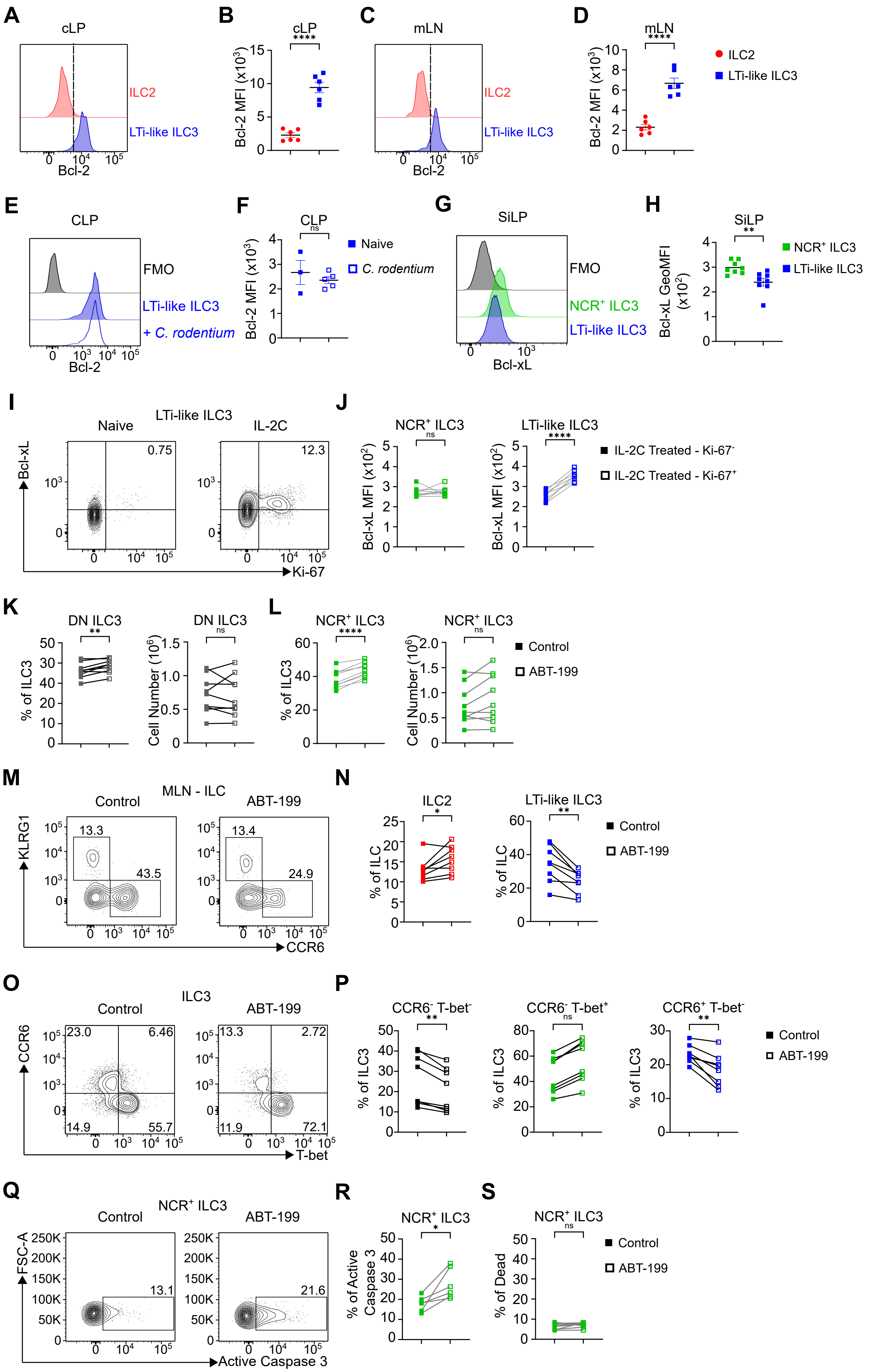
A
cLP
ILC2
LTi-like ILC3
Bcl-2
B
cLP
****
Bcl-2 MFI (x10³)
C
mLN
ILC2
LTi-like ILC3
Bcl-2
D
mLN
****
Bcl-2 MFI (x10³)
ILC2
LTi-like ILC3
E
CLP
FMO
LTi-like ILC3
+ C. rodentium
Bcl-2
F
CLP
ns
Bcl-2 MFI (x10³)
Naive
C. rodentium
G
SiLP
FMO
NCR+ ILC3
LTi-like ILC3
Bcl-xL
H
SiLP
**
Bcl-xL GeoMFI (x10²)
NCR+ ILC3
LTi-like ILC3
I
LTi-like ILC3
Naive
IL-2C
Bcl-xL
0.75
12.3
Ki-67
J
NCR+ ILC3
ns
LTi-like ILC3
****
Bcl-xL MFI (x10²)
IL-2C Treated - Ki-67-
IL-2C Treated - Ki-67+
K
DN ILC3
**
DN ILC3
ns
% of ILC3
Cell Number (10⁶)
L
NCR+ ILC3
****
NCR+ ILC3
ns
% of ILC3
Cell Number (10⁶)
Control
ABT-199
M
MLN - ILC
Control
ABT-199
KLRG1
13.3
43.5
13.4
24.9
CCR6
N
ILC2
*
LTi-like ILC3
**
% of ILC
Control
ABT-199
O
ILC3
Control
ABT-199
CCR6
23.0
6.46
14.9
55.7
13.3
2.72
11.9
72.1
T-bet
P
CCR6- T-bet-
**
CCR6- T-bet+
ns
CCR6+ T-bet-
**
% of ILC3
Control
ABT-199
Q
NCR+ ILC3
Control
ABT-199
FSC-A
13.1
21.6
Active Caspase 3
R
NCR+ ILC3
*
% of Active Caspase 3
S
NCR+ ILC3
ns
% of Dead
Control
ABT-199

Figure S7. Bcl-2 inhibition has little effect on NCR$^+$ ILC3 survival. **(A-D)** Bcl-2 expression in ILC2 and LTi-like ILC3 in cLP and mLN of naïve animals, and **(E-F)** in cLP LT-like ILC3 following *C. rodentium* infection. **(G-H)** Representative histogram and MFI quantification of Bcl-xL in NCR$^+$ ILC3 and LTi-like ILC3 from naïve SiLP. **(I-J)** Bcl-X_L in Ki-67$^-$ and Ki-67$^+$ NCR$^+$ ILC3 and LTi-like ILC3 in IL-2C treated mice. **(K and L)** Frequency and cell number of siLP DN ILC3 and NCR$^+$ ILC3 +/- ABT-199 treatment ex vivo. **(M and N)** Frequency of mLN ILC2 and LTi-like ILC3 +/- ABT-199 treatment ex vivo. **(O-P)** Representative histograms of CCR6 versus T-bet expressing ILC3 in SiLP cells +/- ABT-199 treatment ex vivo. **(Q-S)** Frequency of active caspase 3 expression (Q and R) and live/dead acquisition (S) in NCR$^+$ ILC3 in SiLP +/- ABT-199 treatment ex vivo. ILC2 and ILC3 subsets in panels A-J gated as in Figure S1A, panels Q-S gated as in Figure S1B. Data shown as mean +/- SEM (B-H) or individual data points (I-S) and represent two (B, F, H, J, R, S) or three independent experiments (D, K, L, N, P) (n = 5 – 9). Numbers in flow plot indicate percentage of cells in the respective gate. Data in panels J, K, L, N, P, R and S represent paired samples from same animals with or without treatment. *P< 0.05, **P<0.01, ***P<0.001, ****P<0.0001 using unpaired t-test (B-H) or paired t-test (J-S).

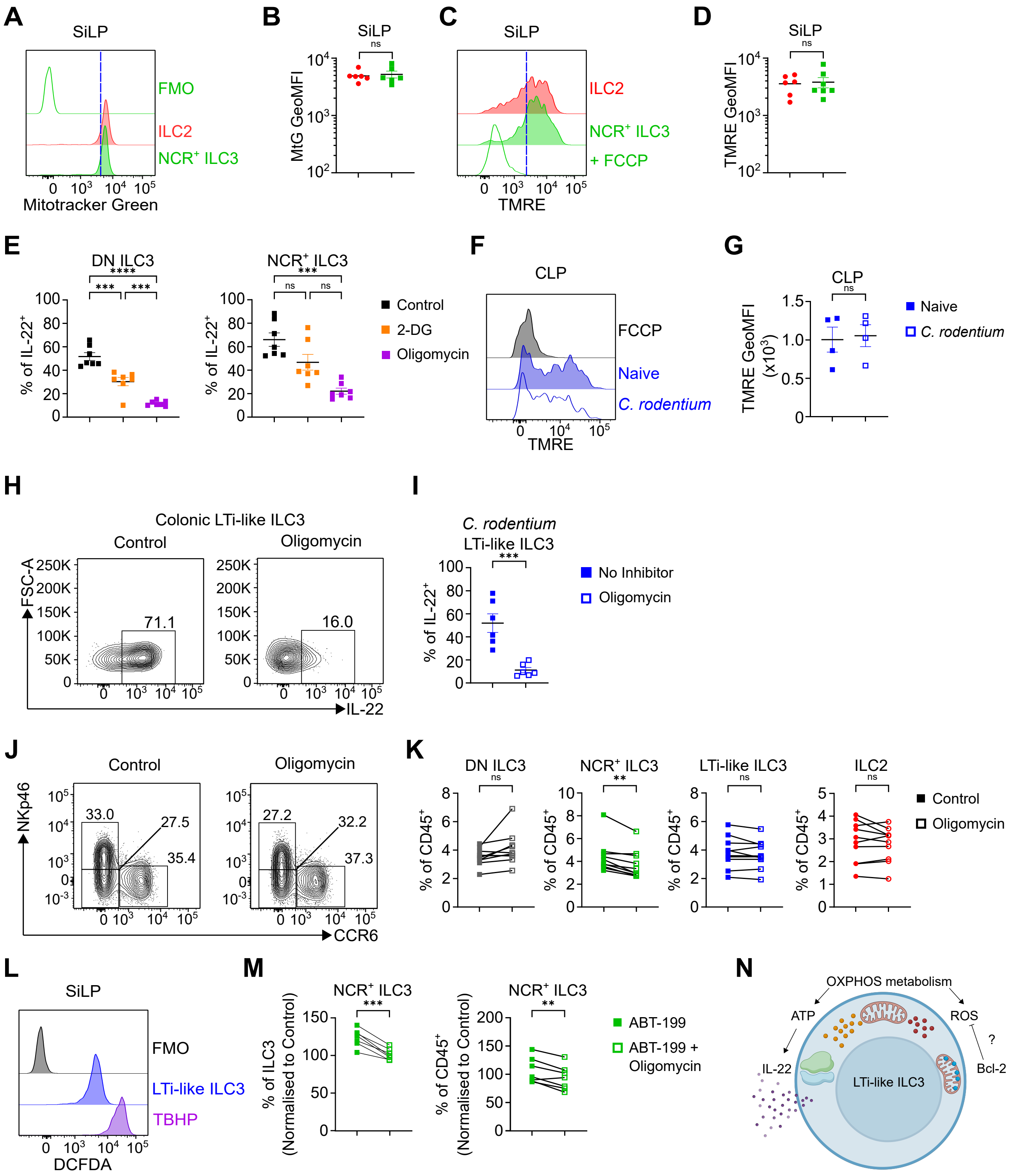

Figure S8. Intracellular balance between metabolism and stress in ILC3. **(A-B)** Representative histogram of mitochondrial mass and MFI in ILC2 and NCR$^+$ ILC3 in SiLP (Dashed blue line represents LTi-like ILC3 average mitochondrial mass). **(C-D)** Representative histogram of TMRE staining and MFI quantification in ILC2 and NCR$^+$ ILC3 in SiLP (Dashed blue line represents LTi-like ILC3 average TMRE intensity). **(E)** IL-1β/-23 induced IL-22 production +/- 2-DG or Oligomycin treatment *ex vivo* in siLP DN ILC3 and NCR$^+$ ILC3. **(F-G)** Representative histogram of TMRE staining and MFI quantification in colonic LTi-like ILC3 from naïve and *C. rodentium* infected mice. **(H-I)** Representative plots and quantification of the percentage of IL-22$^+$ colonic LTi-

like ILC3 from *C. rodentium* infected mice with or without treatment with oligomycin. **(J-K)** Representative plots and quantification of the percentage of siLP ILC subsets in naïve cells cultured *ex vivo* with or without oligomycin. **(L)** Representative histogram of cellular ROS (DCFDA) in DCFDA FMO and LTi-like ILC3 +/- tert-Butyl hydroperoxide (TBHP). **(M)** Normalised frequency of NCR^+ ILC3 treated with ABT-199 +/- Oligomycin treatment in SiLP. **(N)** Cartoon detailing the balance of metabolic stress in LTi-like ILC3 to facilitate function and survival. Panels A-D, F-G and L gated as in Figure S1B, panels E, H-K and M as in Figure S1A. Data shown as mean +/- SEM and individual data points and represent two (E, G) or three independent experiments (B, D, I, K, M) (n = 4-9). Data in K and M represent paired samples from same animals with or without treatment. *P< 0.05, **P<0.01, ***P<0.001, ****P<0.0001 using unpaired t-test (B, D, G, I), paired t-test (K, M) or Dunn's multiple comparisons test (E).
